# The interplay between *Acinetobacter baumannii* ZigA and SltB promotes zinc homeostasis and cell envelope integrity

**DOI:** 10.1128/iai.00422-24

**Published:** 2025-01-23

**Authors:** Jeanette M. Critchlow, Juan P. Barraza, Matthew J. Munneke, Evan Krystofiak, Erin R. Green, Eric P. Skaar

**Affiliations:** 1 Microbe-Host Interactions Training Program, Vanderbilt University School of Medicine 12327 , Nashville, Tennessee, USA; 2 Department of Pathology Microbiology and Immunology, Vanderbilt University Medical Center 204907 , Nashville, Tennessee, USA; 3 Vanderbilt Institute for Infection, Immunology, and Inflammation, Vanderbilt University Medical Center 12328 , Nashville, Tennessee, USA; 4 Cell Imaging Shared Resource, Vanderbilt University 5718 , Nashville, Tennessee, USA; 5 Department of Microbiology, University of Chicago 456539 , Chicago, Illinois, USA; University of Pennsylvania, Philadelphia, Pennsylvania, USA

**Keywords:** ZigA, SltB, zinc, *Acinetobacter baumannii*

## Abstract

*Acinetobacter baumannii* is an opportunistic human pathogen that acquires nutrient metals from the vertebrate host amid infection. During zinc (Zn) scarcity, *A. baumannii* upregulates the expression of the predicted Zn metallochaperone, *zigA*. Loss of *zigA* compromises fitness during Zn deficiency, highlighting its role in this condition. To assess the contribution of ZigA to Zn-deficient *A. baumannii*, a multiparallel transposon sequencing and genetic interaction mapping approach was used. Transposon insertions in *A1S_3027*, encoding a predicted soluble lytic transglycosylase that tailors the bacterial cell wall, were enriched in the Zn-starved Δ*zigA* transposon library. Based on previous studies as well as structural and sequence homology, we designated A1S_3027 as soluble lytic transglycosylase B (SltB). Further analyses revealed that inactivating *sltB* rescued Δ*zigA* fitness defects during Zn starvation. An *A. baumannii* Δ*zigA*Δ*sltB* mutant demonstrated altered cell envelope structures and increased cellular permeability, highlighting the roles of ZigA and SltB in maintaining cell envelope integrity. Furthermore, these mutants exhibited heightened resistance to β-lactam antibiotics and other cell wall-targeting agents. Alterations in cell envelope integrity in the Δ*zigA*Δ*sltB* mutant improved fitness in a murine pneumonia infection model, emphasizing the contribution of ZigA and SltB to *A. baumannii* pathogenesis. This study elucidates how functional interactions between ZigA and SltB modulate cell envelope integrity and pathogenesis of *A. baumannii* during Zn depletion. These findings reveal an interplay between metal homeostasis and cell envelope integrity, offering insights into how *A. baumannii* ZigA contributes to these critical cellular processes.

## INTRODUCTION

*Acinetobacter baumannii* is a Gram-negative, opportunistic human pathogen that has emerged as a leading cause of healthcare-associated infections and outbreaks (1, 2). The increase in *A. baumannii* infections, particularly in hospitals, is largely attributed to intrinsic antibiotic resistance mechanisms, therefore associated with worse clinical outcomes (3, 4). The widespread incidence of multi-drug-resistant infections has prompted the World Health Organization to designate *A. baumannii* as a priority pathogen requiring the development of innovative therapeutics (5).

The bacterial cell envelope serves as a barrier against environmental onslaughts. The Gram-negative cell envelope consists of two membranes separated by a periplasmic region containing peptidoglycan. Peptidoglycan is a heteropolymer of elongated glycan chains of alternating N-acetylglucosamine (GlcNAc) and N-acetylmuramic acid (MurNAc) linked by β1-4 bonds. The MurNAc-GlcNAc disaccharide is further stabilized by short pentapeptides attached to MurNAc and typically crosslinked at the carboxyl group of D-alanine in position four (6–10). Peptidoglycan assembly requires dozens of essential genes, many of which require metals for their function (11–14). Nutrient metals, like zinc (Zn), are vital across all Kingdoms of life. Insufficient or excess uptake of Zn leads to reduced growth and cell death. Zn functions in protein stability and enzymatic reactions, as well as in limiting oxidative protein damage, underscoring the importance of cellular mechanisms that maintain Zn homeostasis (15, 16). The essentiality of Zn is exploited by the vertebrate immune system, which decreases metal bioavailability to *A. baumannii* in a process known as “nutritional immunity” (17, 18). Vertebrate sequestration of Zn, coupled with the requirement of Zn for bacterial survival, emphasizes the necessity for *A. baumannii* to utilize strategies to combat Zn starvation.

Managing bioavailable Zn is predicted to require dedicated metalloproteins, which receive metal from metallochaperones in a hierarchical fashion (19). The G3E P-loop GTPase superfamily, specifically COG0523 proteins, is predicted to function as metallochaperones or specialized intracellular proteins that facilitate the specific deposition of metal into metalloproteins (20). The assignment of COG0523 proteins as metallochaperones is supported by studies into eukaryotic ZNG1 and suggested by work on the bacterial proteins ZigA and ZagA (21–24). In response to Zn starvation, *A. baumannii* significantly upregulates the expression of the predicted metallochaperone, ZigA, a COG0523 protein crucial for growth in this condition (25). The response of *zigA* to Zn starvation is regulated by the zinc uptake repressor Zur, which also controls the expression of mechanisms for acquiring zinc from labile histidine-bound zinc pools, as well as high-affinity transport systems like ZnuABC (25–28). However, the impact of Zn deficiency on other aspects of *A. baumannii* physiology remains unknown. Given the contribution of ZigA to *A. baumannii* survival during Zn limitation and its predicted function as a Zn metallochaperone, we sought to identify functional interactions with ZigA to understand how ZigA supports cellular survival during Zn starvation.

To understand the mechanisms by which ZigA maintains cellular homeostasis during Zn limitation, this study leveraged multiparallel transposon sequencing (Tn-seq) and genetic interaction mapping to identify genes that exhibit strong interactions with *zigA*. Transposon insertions in *A1S_3027* were enriched in the absence of *zigA* under Zn-limiting conditions, indicating that the loss of A1S_3027 confers decreased fitness in a Δ*zigA* background compared to the loss of A1S_3027 in a wild-type (WT) background. Inactivating *A1S_3027* suppresses the fitness burden of Δ*zigA* during Zn starvation. *A1S_3027,* annotated as *slt*, encodes a predicted soluble lytic transglycosylase for cell wall recycling and signaling, cell envelope maintenance, and membrane protein insertion (29–32). Previous studies have shown that *A1S_3027* and *A1S_0178* are predicted soluble lytic transglycosylases, which play a role in virulence and antibiotic susceptibility in *A. baumannii* (8, 33–36). Based on this similarity and structural sequence comparisons to known lytic transglycosylase structures, we have named A1S_3027 as soluble lytic transglycosylase B (SltB). This research uncovers the role of ZigA in maintaining cell envelope integrity and managing Zn homeostasis in *A. baumannii*, providing new insights into functional interactions with SltB and their combined contribution to bacterial pathogenesis.

## RESULTS

### Identification of essential genes in a zinc-starved *zigA* mutant transposon library

Genetic interaction mapping, which can be inferred from transposon sequencing methodologies, has been employed to study gene essentiality and fitness across many bacterial species (37, 38). This technique identifies gene interactions by comparing insertion profiles and fitness across different strain backgrounds and mutations (39). To identify transposon mutants whose insertional frequency and fitness are altered by genetically inactivating *zigA*, genetic interaction mapping was employed to further understand the importance of ZigA during Zn depletion. To do this, WT *A. baumannii* 17978 and Δ*zigA* transposon libraries were generated and grown in LB media ± tetrakis-(2-pyridylmethyl)ethylenediamine (TPEN). Transposon junctions from the libraries were amplified and subjected to massively parallel sequencing of transposon-genome junctions (Tn-seq). The insertions in each library were mapped to approximately 100,000 unique chromosomal positions.

**Fig 1:**
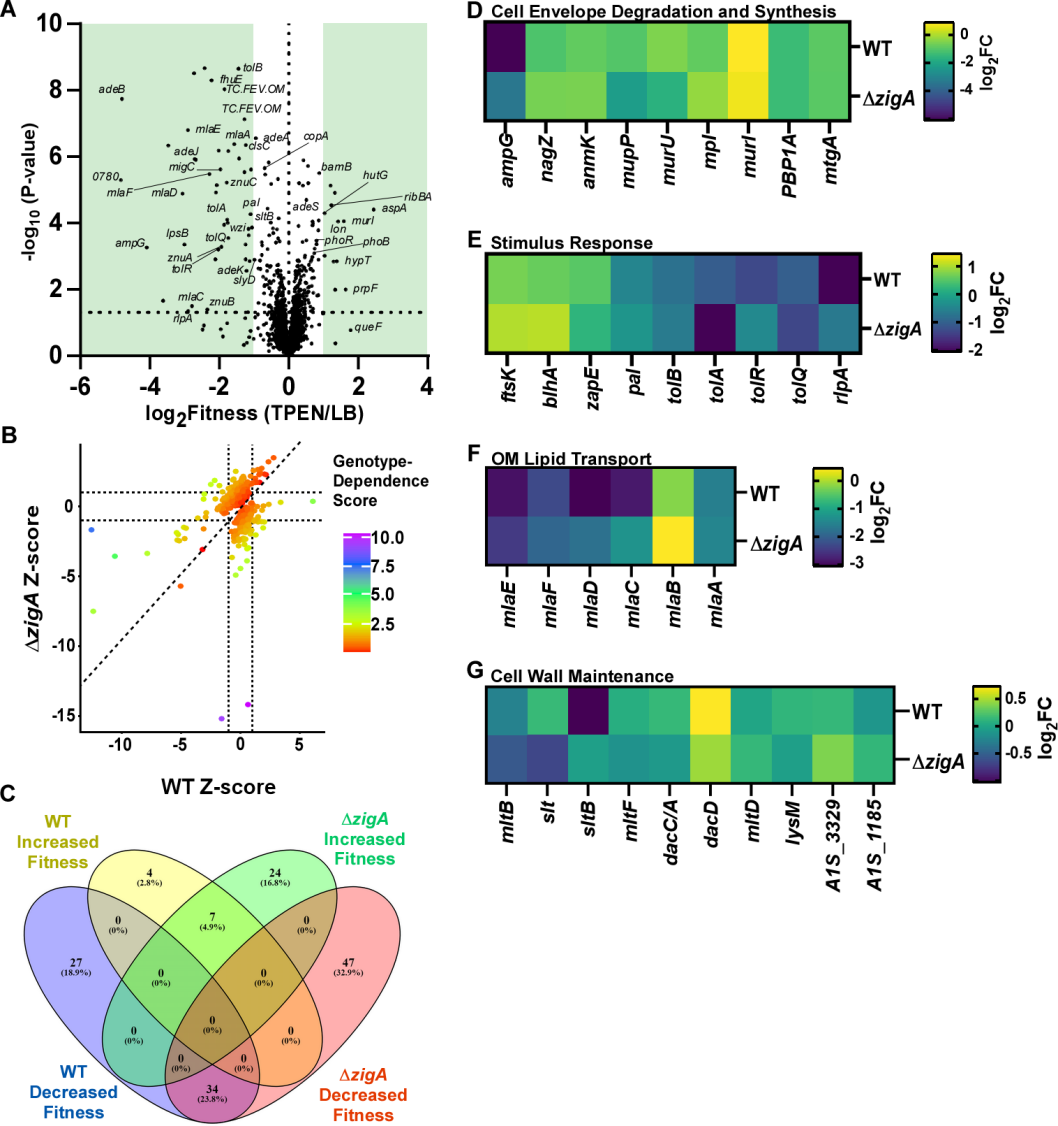
ZigA contributes to critical cellular pathways during zinc starvation. (**A**) Volcano plot of the relative fitness in the ATCC 17978 WT library comparing TPEN treatment to rich media. Genes with a fitness of ±1.5 were considered to be differentially fit and are highlighted in green. (**B**) *Z*-score of Δ*zigA* library compared to the WT, where the slope of the line indicates genes whose fitness was impacted by genetically inactivating *zigA*. (**C**) A Venn diagram that depicts differentially fit genes whose fitness was influenced by TPEN treatment, genotype, or both. (**D–G**) A gene ontology term enrichment analysis of (**D**) cell envelope degradation and synthesis, (**E**) stimulus response, (**F**) outer membrane (OM) lipid transport, or (**G**) cell wall maintenance. Heat maps show the log2 fold change (log_2_FC) in reads when comparing the TPEN-treated media to rich media. These data are further compared by genotype (WT vs Δ*zigA*).

To assess the mediators of Zn starvation, genes with altered insertional frequencies and fitness were first identified in the WT library (Fig. 1A; Table S3). Genes with previously described roles in cellular Zn uptake, such as *znuB* (−3.43 TPEN fitness, 3.16 TPEN/LB fitness) and *znuC* (−2.19, 5.44), showed reduced fitness (28). Similarly, genes related to iron acquisition, including *bauA* (–1.28, –2.25), *bauB* (−2.5, 5.3), and *bauC* (−2.78, 3.02), had attenuated fitness (40). An *A. baumannii* COG0523 ortholog of *zigA*, *A1S_0934* (–1.36,–2.2), also displayed reduced fitness during Zn depletion, supporting the hypothesis that the COG0523 enzyme family contributes to cellular survival during Zn stress (41). Genes linked to cell envelope maintenance, biosynthesis, signaling, transport, or recycling were uniformly less fit and include *ampG* (−6.34, 2.8), *adeB* (−5.48, 8.04), *mlaD* (−3.12, 47.1), *tolR* (−4.5, 1.82), and *lpxL* (−4.54, 1.63), with *A1S_0780* (−7.4, 2.88), involved in Fe-S cluster formation, being the least fit (Fig. 1A; Tables S3 and S5). These findings highlight the multiple essential cellular processes that respond to Zn depletion.

To identify genes that exhibit strong interactions with *zigA*, total insertional reads were compared between the WT and Δ*zigA* libraries. Genes with fewer than 50 reads in either background were excluded from analysis, including shared and unique essential genes in the WT and Δ*zigA* libraries (Fig. S1A). A log2 fold change (Log2FC) of 1.5 in either direction indicated potential genetic interactions with *zigA*. These interactions were further analyzed by comparing *Z*-scores—statistical measures of deviation from the mean—between the libraries to assess genotype dependence (Fig. 1B). This approach identified 71 hits that were differentially fit when *zigA* was absent in the Tn library and Zn was depleted (Fig. 1C; Fig. S1B; Table S4). An additional 31 hits in the WT library exhibited differential fitness in Zn-deplete conditions, irrespective of *zigA*, indicating genotype specificity (Fig. 1C; Tables S4 and S5).

Given that ZigA is important for the growth of *A. baumannii* during Zn depletion, we hypothesized that genes within key cellular pathways involved in cellular replication would demonstrate strong interactions with *zigA*. Conducting a gene ontology term enrichment analysis revealed that pathways involved in energy production, the flow of genetic information, and the cell exterior are affected by *zigA* inactivation (Fig. S2A). As previous literature has outlined the importance of Zn in the synthesis and maintenance of the cell envelope, we chose to interrogate the relevant pathways within *A. baumannii* (12, 28, 42, 43). These include genes involved in cell envelope degradation (Fig. 1D), stimulus response (Fig. 1E), outer membrane lipid transport (Fig. 1F), cell wall maintenance (Fig. 1G), phosphate utilization (Fig. S2B), the rod system (Fig. S2C), and antibiotic resistance (Fig. S2D). Of the 150 genes with significant differential fitness between the Δ*zigA* (TPEN/LB) and WT (TPEN/LB) libraries, approximately 30% were linked to these cellular processes, highlighting their potential as candidates for further study.

Genetic interactions involving *zigA* highlight functional associations with proteins critical for cell envelope maintenance. These interactions also serve as a phenotypic characterization that underscores the importance of *zigA* in bacterial physiology during Zn depletion. One type of genetic interaction, known as suppression, occurs when a second genomic perturbation results in compensation for the detrimental effects of a primary mutation (44). This interaction type can occur between closely related genes, either within the same protein complex or pathway, or in an alternate pathway affecting the same cellular process (44). Genes exhibiting genetic suppression with *zigA* during Zn depletion, where fitness is higher in the Δ*zigA* library compared to WT, include those involved in Zn uptake (*znuA, znuB,* and *znuC*), lipid transport (*mlaB, mlaC, mlaD,* and *mlaF*), histidine catabolism and uptake (*hutG* and *hisQ*), lipooligosaccharide biosynthesis (*lpsB, lpsC, lpxL,* and *lpxO*), capsule production (*wzi* and *gtr9*), cell wall synthesis and degradation (*ampG, murU,* and *elsL*), and cell wall maintenance (*A1S_3027, rodA,* and *lysM*) (Tables S4 and S5). Collectively, these findings identified genes whose functional interactions with ZigA influence bacterial fitness, revealing a pathway involved in cell envelope maintenance.

### Inactivating *sltB* compensates for the loss of *zigA*

**Fig 2:**
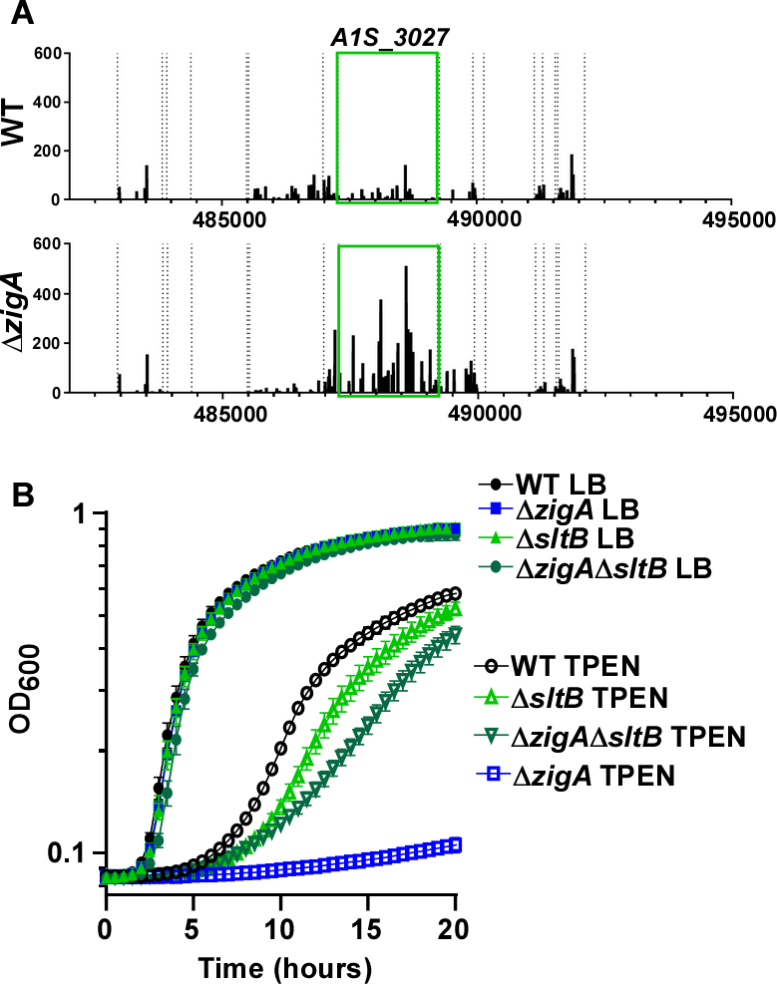
A mutation in *sltB* compensates for the loss of *zigA* during zinc depletion. (**A**) Transposon mutant mapping in *A. baumannii* of *sltB* during Zn deficiency. Highlighted area indicates the coding region. (**B**) WT, Δ*zigA*, Δ*sltB*, and Δ*zigA*Δ*sltB* were grown LB ± 40 µM TPEN with OD_600_ monitored over time.

*A1S_3027*, annotated as *slt*, encodes a soluble lytic transglycosylase involved in cell wall recycling, signaling, and envelope maintenance (29–36). Transposon insertions in *A1S_3027* were enriched in the Δ*zigA* background following growth in TPEN, suggesting improved fitness (Fig. 2A). AlphaFold2 analysis revealed structural conservation between A1S_3027 and *Pseudomonas aeruginosa* Slt (Fig. S3A) and identified essential lytic transglycosylase domains, including a signaling peptide sequence, transglycosylase super helical domain, super helical linker, and a soluble lytic transglycosylase domain, which support the function of A1S_3027 as a lytic transglycosylase (Fig. S3B) (45). These and previous findings suggest that A1S_3027 encodes a second soluble lytic transglycosylase in *A. baumannii*, leading to the designation of A1S_3027 as SltB (8).

To interrogate ZigA-SltB functional interactions, we generated Δ*zigA*, Δ*sltB,* and Δ*zigA*Δ*sltB* mutants and monitored growth in LB media supplemented with 40 µM TPEN. Both Δ*zigA* and Δ*sltB* were sensitized to Zn depletion with Δ*zigA* exhibiting more pronounced growth defects (Fig. 2B; Tables S1 and S2). The Δ*zigA* fitness defect during Zn restriction was partially alleviated in the absence of *sltB*, consistent with genetic suppression and indicating that inactivating *sltB* may relieve the toxic effect of mutating *zigA* directly or through inducing a compensatory pathway. These fitness defects were fully rescued by the expression of *zigA, sltB*, or both in *cis* (Fig. S3C) and equimolar ZnCl_2_ supplementation (Fig. S3D). These data highlight that inactivating *sltB* alleviates the fitness burden of mutating *zigA* during Zn depletion, which may influence the integrity of the cell envelope.

### A *zigA-sltB* mutant has altered cell envelope structure and permeability

**Fig 3:**
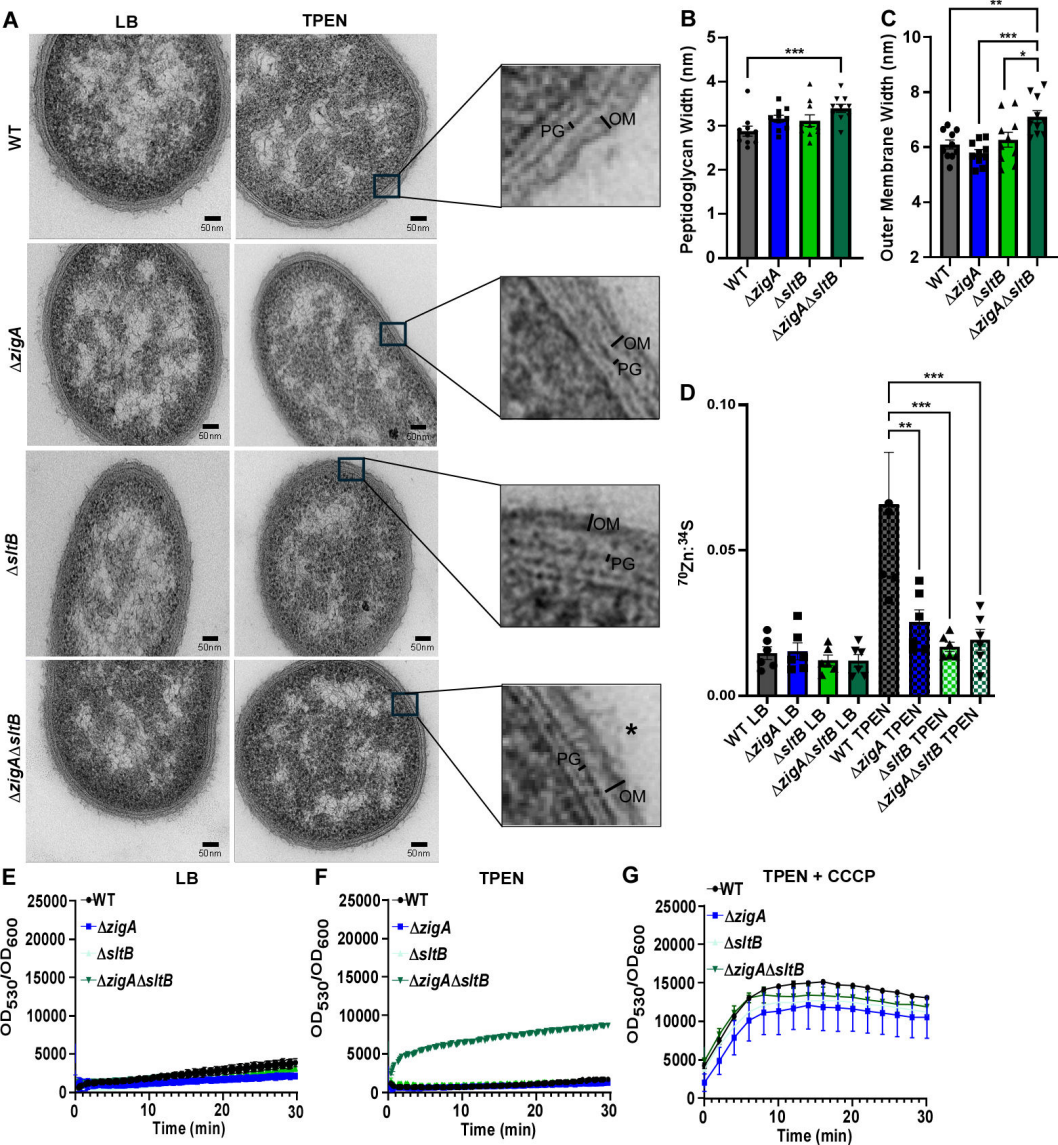
ZigA-SltB functional interactions significantly contribute to both the structure and permeability of the cell envelope. (**A**) Electron micrographs of WT and Δ*zigA*Δ*sltB* in LB ± 40 µM TPEN. Amplifications of the cell envelope are depicted. Peptidoglycan (PG) and the outer membrane (OM) are further indicated. Cellular extrusions are marked with an asterisk. (**B**) Peptidoglycan and (**C**) outer membrane width of cells was assessed using ImageJ software. Each *n* is an average of 100 cells per genotype and per condition. *P < 0.05, **P < 0.01, ***P < 0.001, and ****P < 0.0001 by one-way ANOVA. (**D**) ^70^Zn uptake was quantified by inductively coupled plasma mass spectrometry and normalized to ^34^S for WT, Δ*zigA*, Δ*sltB*, and Δ*zigA*Δ*sltB* ± 40 µM TPEN. **P < 0.01 and ***P < 0.001 by one-way ANOVA with Šídák’s multiple comparisons test. Ethidium bromide uptake following early stationary growth in (**E**) LB, (**F**) 40 µM TPEN, and (**G**) 40 µM TPEN + CCCP.

As SltB likely functions as a soluble lytic transglycosylase that cleaves the glycosidic bond between N-acetylglucosamine (GlcNAc) and N-acetylmuramic acid (MurNAc) dimers, we hypothesized that inactivation of *sltB* could alleviate the fitness impact of mutating *zigA* through altering the cell wall. To visualize the impact of *zigA* and *sltB* mutations on the cell morphology, cell envelope structures of WT, Δ*zigA*, Δ*sltB*, and Δ*zigA*Δ*sltB* were evaluated in LB- or Zn-depleted media using transmission electron microscopy (Fig. 3A; Fig. S4A). These analyses revealed that Δ*zigA*Δ*sltB* exhibited distended peptidoglycan (Fig. 3B) and outer membrane (Fig. 3C) structures during Zn depletion, with no changes observed under Zn-replete conditions (Fig. S4B through D). This distention, not observed in either Δ*zigA* or Δ*sltB*, is a phenotypic characterization of ZigA-SltB functional interactions. While alterations were evident in the outer membrane and peptidoglycan, there were no discernable differences to the inner membrane (Fig. S4C), suggesting that the absence of *sltB* may alter only specific portions of the cell envelope under these conditions.

We predicted that the abnormal cell wall and envelope structures reveal the importance of the ZigA-SltB functional interactions in facilitating Zn uptake. To test this hypothesis, WT, Δ*zigA*, Δ*sltB,* and Δ*zigA*Δ*sltB* were grown in LB ± 40 µM TPEN and pulsed with a rare isotope of Zn, ^70^Zn, after which intracellular metals were quantified using inductively coupled plasma mass spectrometry (ICP-MS). Following TPEN-mediated Zn starvation, WT demonstrated increased ^70^Zn uptake relative to the LB control (Fig. 3D). All mutant strains exhibited reduced Zn uptake compared to WT, suggesting that while ZigA and SltB each contribute to Zn uptake, this effect is not due to genetic interactions between them. This pattern was observed when quantifying naturally occurring abundant isotopes of ^66^Zn (Fig. S5A) but not ^56^Fe (Fig. S5B), suggesting a specificity for Zn. The Δ*zigA*Δ*sltB* double mutant suppresses the Δ*zigA* phenotype during Zn depletion while exhibiting similar Zn uptake as Δ*sltB*, suggesting loss of *sltB* bypasses the *zigA* phenotype via an alternative pathway.

As Zn uptake does not contribute to the altered peptidoglycan architecture in the Δ*zigA*Δ*sltB* mutant, we hypothesized that inactivating *zigA* and *sltB* alters cell envelope permeability. To test this, we quantified ethidium bromide (EtBr) diffusion rates across WT, Δ*zigA*, Δ*sltB*, and Δ*zigA*Δ*sltB* cellular envelopes by monitoring the fluorescence emitted through EtBr-DNA interactions (8). While the fluorescence remained unchanged in LB (Fig. 3E), there was an increase in fluorescence in the Δ*zigA*Δ*sltB* mutant compared to WT grown in Zn-deplete media, indicating increased cell envelope permeability (Fig. 3F). This increase in permeability was complemented by integrating *zigA* and *sltB* in *cis* (Fig. S5C). To assess if the difference in EtBr accumulation is due to deficiencies in efflux in the Δ*zigA*Δ*sltB* mutant, EtBr accumulation was measured across WT, Δ*zigA*, Δ*sltB*, and Δ*zigA*Δ*sltB* strains treated with the proton motive force uncoupler carbonyl cyanide m-chlorophenylhydrazone (CCCP). The use of CCCP equalized EtBr accumulation across all strains, indicating that this phenotype may be partly due to deficient efflux in Δ*zigA*Δ*sltB* (Fig. 3G). These findings underscore that ZigA-SltB functional interactions influence cell envelope stability and peptidoglycan architecture, collectively affecting the sensitivity of *A. baumannii* to Zn depletion.

### *zigA-sltB* mutants alter *A. baumannii* pathophysiology

**Fig 4:**
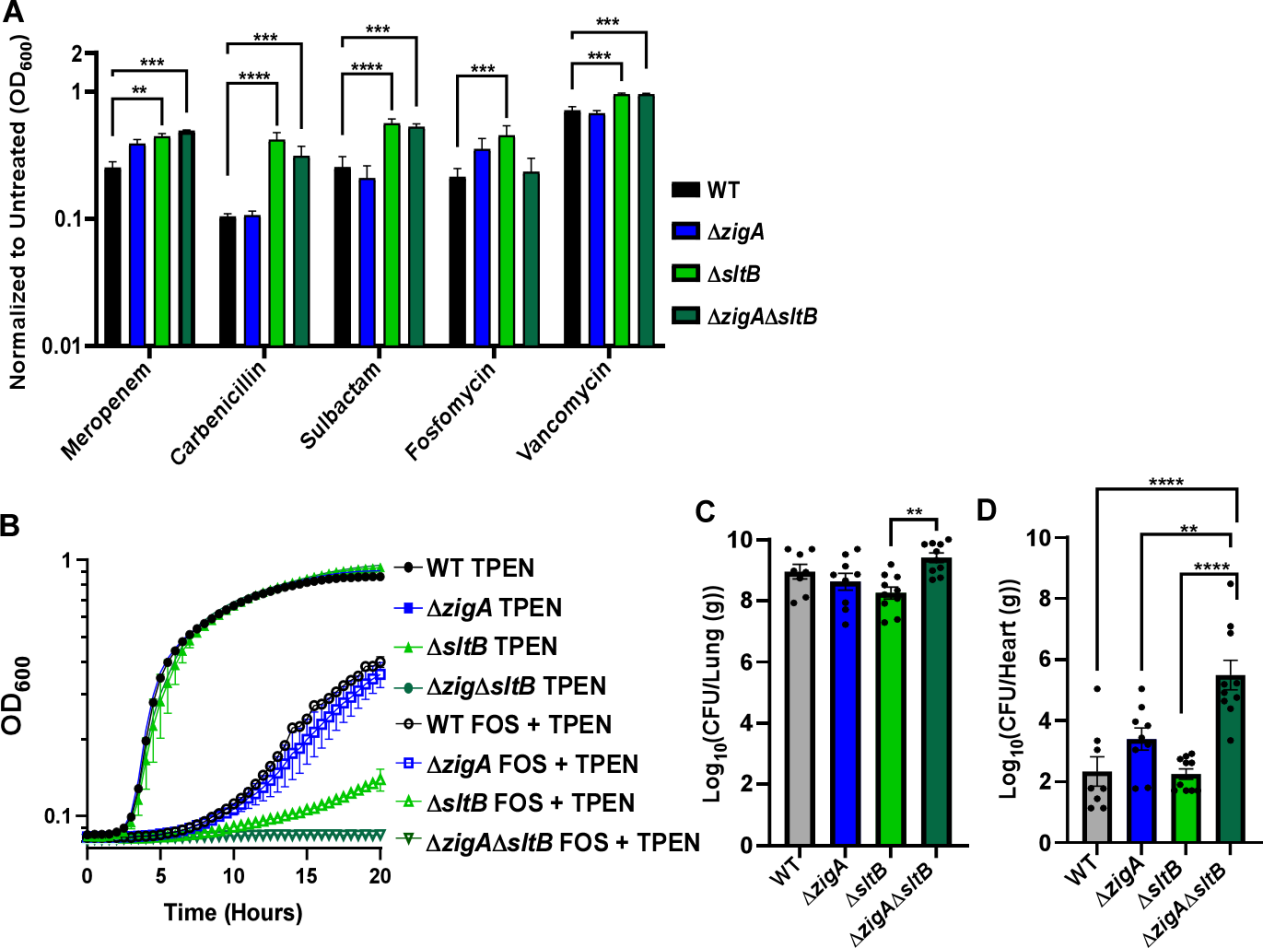
ZigA-SltB functional interactions alter *A. baumannii* antibiotic susceptibility and pathogenesis. (**A**) Growth of WT, Δ*zigA*, Δ*sltB*, and Δ*zigA*Δ*sltB* in LB for 13 hours ± 1.25 µg/mL meropenem, 7.57 µg/mL carbenicillin, 0.5 µg/mL sulbactam, 128 µg/mL fosfomycin, or 32 µg/mL vancomycin. **P < 0.01, ***P < 0.001, and ****P < 0.0001 as determined by two-way ANOVA with Tukey multiple comparisons test (*n* = 9 biological replicates per genotype per group). (**B**) Growth of WT, Δ*zigA*, Δ*sltB*, and Δ*zigA*Δ*sltB* in LB ± 64 mg/mL fosfomycin + 20 µM TPEN assessed using OD_600_ for 20 hours WT, Δ*zigA*, Δ*sltB*, and Δ*zigA*Δ*sltB* bacterial burdens recovered from the (**C**) lungs or (**D**) heart at 36 hours post-infection. **P < 0.01 and ****P < 0.0001 as determined by one-way ANOVA with Šídák’s multiple comparisons test (*n* = 8–10 biological replicates per genotype).

Since inactivating *zigA* and *sltB* affects peptidoglycan architecture, we hypothesized that Δ*zigA*Δ*sltB* mutants would have increased antibiotic sensitivity. To test this, we grew WT, Δ*zigA*, Δ*sltB*, and Δ*zigA*Δ*sltB* strains in LB treated ± meropenem, carbenicillin, sulbactam, fosfomycin, or vancomycin. Both Δ*sltB* and Δ*zigA*Δ*sltB* mutants exhibited decreased susceptibility to β-lactam antibiotics (meropenem and carbenicillin) compared to WT cells, but not relative to each other, indicating that SltB mediates antibiotic resistance independently of ZigA (Fig. 4A). Additional decreases in susceptibility were observed in the Δ*sltB* and Δ*zigA*Δ*sltB* mutants with non-β-lactam antibiotics targeting cell wall biosynthesis, maintenance and recycling, including sulbactam, fosfomycin, and vancomycin. However, modulation of Zn using a non-inhibitory TPEN treatment combined with fosfomycin demonstrated increased sensitivity, highlighting SltB mediates fosfomycin sensitivity in Zn-deplete, but not replete, conditions (Fig. 4B).

Given that invading bacterial pathogens experience Zn insufficiency *in vivo*, we investigated the contribution of ZigA-SltB functional interactions to *A. baumannii* pathogenesis using a murine pneumonia model of infection (8, 46–48). Eight-week-old female C57BL/6J mice were infected intranasally with the WT, Δ*zigA*, Δ*sltB*, and Δ*zigA*Δ*sltB* strains and monitored over time. At 36 hours post-infection, mice were humanely euthanized, and organs (lungs, spleens, kidneys, livers, and hearts) were collected for bacterial burden enumeration. While no fitness alteration was observed in the lungs (Fig. 4C), the Δ*zigA*Δ*sltB* mutant demonstrated significantly higher bacterial burdens and survival in the heart (Fig. 4D), with trends for increased burden in the liver (Fig. S6A), spleen (Fig. S6B), and kidneys (Fig. S6C). Taken together, these data indicate that while ZigA-SltB functional interactions influence cellular permeability, their disruption leads to a greater dissemination of *A. baumannii* in a murine pneumonia model, thus altering the pathogenicity of a clinically relevant pathogen.

## DISCUSSION

In this study, we determined that the *A. baumannii* COG0523 and predicted metallochaperone ZigA play a role in cell envelope integrity and survival during Zn depletion. Genotypic and phenotypic analyses showed that a Δ*zigA* fitness defect in Zn-deficient media is suppressed in the absence of *sltB*, a predicted lytic transglycosylase, indicating that *sltB* alleviates the fitness burden of inactivating *zigA* in this condition. ZigA-SltB functional interactions altered cell envelope structures and permeability. Mutants lacking *sltB* showed decreased susceptibility to β-lactam antibiotics, although Zn modulation combined with antibiotics targeting cell wall biogenesis increased sensitivity, indicating that this is a functionally independent activity. In a murine pneumonia model, a Δ*zigA*Δ*sltB* mutant exhibited higher levels of dissemination to the heart relative to a WT strain or either single mutant, revealing ZigA-SltB functional interactions alter the survival of *A. baumannii* in the vertebrate host.

ZigA is a COG0523 subfamily GTPase that functions in a Zn-dependent manner and potentially contributes to metallocenter formation (22, 23, 49–52). Similar to *Escherichia coli* YeiR, ZigA expression is stimulated by and required for growth during Zn starvation (22, 23, 49–52). This study determined that genetically inactivating *sltB* alleviated the fitness cost of a *zigA* mutation during Zn deficiency (Fig. 2A and B; Fig. S3A through D). While specific metalation of metalloenzymes by ZigA may occur, it remains unclear whether the contribution of ZigA to growth in low Zn conditions is direct or indirect via a partner protein. Studies to identify direct biochemical interactions between ZigA and SltB were inconclusive, but we hypothesize that SltB and ZigA act on the same pathway or protein. SltB is predicted to function in the cleavage of β1-4 glycosidic linkages to form 1,6-anhydro-N-acetylmuramic acid (anhMurNAc), which is transported to the cytoplasm by AmpG to be recycled (8, 29–36). In line with our prediction that ZigA and SltB function in the same pathway, *ampG* was identified in our genetic interaction mapping approach as a genetic suppressor of the fitness burden of inactivating *zigA* during Zn deficiency, further supporting that ZigA contributes to this process. Future studies will focus on identifying interaction partners of ZigA.

Cell envelope maintenance is crucial for the pathophysiology of *A. baumannii* (8, 29–36, 53, 54). Zn plays a role in maintaining bacterial cell envelope integrity by stabilizing membrane structures and supporting cell wall biosynthesis (6, 11–14, 28). Indeed, both penicillin-binding protein (PBP2) and Zn-regulated D,D carboxypeptidase A (ZrlA), which are involved in cell wall elongation, bind Zn, highlighting the importance of Zn to this process (12, 14). Lytic transglycosylases, which cleave glycan strands, bind calcium and sodium for their catalytic activity (29, 55). Furthermore, Gram-negative lytic transglycosylases bind Zn for protein stabilization, suggesting that Zn ion coordination contributes to cell wall maintenance, though their exact functions remain unclear (29, 56). The host defense protein calprotectin inhibits *A. baumannii* growth and protects against *A. baumannii* infection in a murine model of pneumonia through chelating Zn with high affinity (47). A Tn-seq screen identified genes important for growth in Zn-deficient conditions, and calprotectin-treated media further revealed that genes involved in Zn uptake (*znuB*), lipooligosaccharide biosynthesis (*lpsB*), and capsule production (*gtr9*) were less fit during Zn deficiency, thus further implicating Zn in cell envelope homeostasis (26).

Given the essential nature of the cell envelope for bacterial survival, functional redundancy is common among cell envelope-modifying enzymes, including lytic transglycosylases (31, 55, 56). *A. baumannii* is predicted to encode two soluble lytic transglycosylases (Slt and SltB) and at least four additional membrane-bound lytic transglycosylases (8, 29–36). Since many *A. baumannii* genes remain unannotated, additional lytic transglycosylases may yet need to be identified. This study determined that the inactivation of *sltB* alleviates the fitness burden of a *zigA* mutation during Zn restriction but does not fully explore the roles of other proteins in the same pathway or other transglycosylases in these processes. Future efforts will explore potential redundant mechanisms maintaining cell envelope integrity.

The bacterial cell envelope protects bacterial pathogens, like *A. baumannii*, against environmental onslaughts, such as osmolarity, pH, antibiotics, and the vertebrate immune system (57–59). Bacteria respond to these challenges through an envelope stress response to maintain cell envelope homeostasis (57). Lytic transglycosylases, like SltB, contribute to this response, and ZigA-SltB functional interactions demonstrate indications of altered cell envelope integrity and permeability (8, 29–36, 57–61). Inactivating *sltB* in the absence of *zigA* leads to increased membrane permeability, possibly due to the loss of the function of SltB in maintaining membrane integrity. We predict that this increase contributes to the envelope stress response observed during Zn restriction, as the compromised membrane may exacerbate the stress experienced by the bacterial envelope under Zn-limited conditions. Inactivating *sltB* in the absence of *zigA* increases membrane permeability, which we predict contributes to the envelope stress response during Zn restriction. We further hypothesize that ZigA contributes to cell envelope biogenesis, recycling, or maintenance, and increased cell envelope permeability compensates for its loss. The mechanism by which the inactivation of *sltB* individually and in tandem with *zigA* contributes to membrane permeability during Zn depletion remains unclear.

Nutrient Zn limitation in bacterial pathogens impairs growth and promotes cell wall remodeling (62, 63). Previous studies have demonstrated that membrane lytic transglycosylase B (MltB) is important for *A. baumannii* during bloodstream infections (33). Lytic transglycosylases also play roles in the pathogenesis of *Neisseria gonorrhoeae* and *Brucella abortus*, but their contributions to infection remain poorly understood (64, 65). In a murine pneumonia model, the Δ*zigA*Δ*sltB* mutant exhibits higher dissemination to the heart, highlighting the importance of ZigA-SltB genetic interactions in *A. baumannii* pathogenesis. These findings are intriguing as the heart may experience decreased Zn sequestration compared to the lungs, allowing for the persistence and dissemination of the Δ*zigA*Δ*sltB* mutant and a trend for increased dissemination of Δ*zigA*. Furthermore, the unique immune environment in the heart compared to the lungs could promote bacterial survival, further increasing the persistence of these mutants.

Our genetic interaction approach suggests that ZigA functionally interacts with both SltB and AmpG. AmpD, a Zn-dependent peptidoglycan recognition protein, cleaves glycan strands from stem peptides for cell wall recycling, acting on anhMurNAc delivered by AmpG (66). Although the mechanism linking *zigA* and *sltB* inactivation to increased pathology remains unclear, we hypothesize that this may involve interactions with AmpD. Inactivation of *ampD* is known to enhance biofilm formation, which could be exacerbated in the absence of *zigA*, potentially leading to prolonged mechanical lung injury (66). Beyond a possible connection with AmpD and biofilm formation, the increase in bacterial burdens in the Δ*zigA*Δ*sltB* mutant may be attributable to alterations in peptidoglycan structure, decreasing immune recognition, and potentially enhancing vascular permeability. Future research will investigate how ZigA-SltB interactions influence tissue-specific colonization.

In summary, this study demonstrates that ZigA and SltB are linked to the growth and survival of *A. baumannii* during Zn depletion. ZigA-SltB functional interactions are important for maintaining cell envelope integrity, permeability, and pathogenesis. These findings emphasize the interplay between metal homeostasis, bacterial fitness, and the cell envelope, suggesting potential therapeutic targets against multidrug-resistant *A. baumannii* infections.

## MATERIALS AND METHODS

### Strain generation

The strains and plasmids used are detailed in Table S1, and primers are listed in Table S2. The Δ*zigA*, Δ*sltB*, and Δ*sltB*Δ*zigA* mutants were generated by allelic exchange, involving PCR amplification of 1,000 bp flanking regions of *A1S_3411* and *_3027* from *A. baumannii* genomic DNA (gDNA) (Table S2). For Δ*sltB* and Δ*sltB*Δ*zigA* mutants, *sltB* was replaced with a kanamycin resistance gene (*aphA*) derived from pUK1. These constructs were cloned into pFLp2 using HIFI assembly. The Δ*zigA* mutant was similarly generated by allelic exchange but remained unmarked. Plasmid transfer into wild-type ATCC 17978 VU *A. baumannii* was achieved via tri-parental conjugation with *E. coli* HB101 containing pRK2013. Selection was performed on Luria Broth Agar (LBA) plates supplemented with 75 µg/mL carbenicillin and 15 µg/mL chloramphenicol. Sucrose selection was then applied to resolve integrated plasmids, and clones were screened by PCR using specific primers. For Δ*sltB* mutants, screening was performed on LBA with kanamycin. Complementation vectors were constructed in pKNOCK (67). The complementation strains (Δ*zigA attTn7::mTn7* (amp^R^)-*zigA*, Δ*sltB attTn7::mTn7*(amp^R^)-*sltB*, and Δ*sltB*Δ*zigA attTn7::mTn7*(amp^R^)-*sltB-zigA*) were constructed by amplifying native promoters and genes from WT ATCC 17978 *A*. *baumannii* into pKNOCK-mTn7-Amp digested with BamHI and KpnI, followed by introduction into respective mutants using mini-Tn7 transposition and four-way mating with *E. coli* containing a helper plasmid (*Escherichia coli* HB101 pRK2013), the transposase (*Escherichia coli* pTNS2), the vectors, and *A. baumannii* as previously described (61). Double complementation was made through amplification and ligation of P*_zigA_-zigA* and P*_sltB_-sltB* in sequential order and then integration into Δ*sltB*Δ*zigA* using mini-Tn7 transposition as described above. Selection for m-Tn7 introduction was performed with carbenicillin as selection and chloramphenicol to counterselect against *E. coli*. All knockout strains were confirmed by whole-genome sequencing, and all complementation constructs were confirmed by Sanger sequencing.

### Transposon sequencing (libraries, screen, and analysis)

To generate the transposon libraries, a vector with a Himar1 transposon, pNJW684 was used to create the transposon mutant pool (68). A previously created WT ATCC 17978 transposon library was used for this screen (69). The Δ*zigA* transposon library was made by mating SM10λpir carrying pJNW684 with Δ*zigA* after overnight growth at 37°C with shaking at 180 rpm. Bacterial cultures were washed three times in 1× PBS before mixing SM10λpir(pJNW684) with WT *A. baumannii* at a 2:1 ratio. The mixtures were plated on LBA plates and allowed to mate for 4 hours at 37°C. Bacteria were then resuspended from plates, and a portion of the mating mixture was spread onto LBA plates supplemented with 40 µg/mL kanamycin and 15 µg/mL chloramphenicol. Approximately 100,000 colonies from the resulting library pools were scraped off the plates, resuspended in a medium containing 20% glycerol, and stored at −80°C.

Transposon library aliquots were back diluted and grown for 3.5 hours and then were used to inoculate into 10 mL LB ± 40 µM TPEN (six biological replicates per condition) and grown for 8 hours at 37°C with shaking at 180 rpm. Bacteria were harvested, washed twice with PBS, pelleted, and stored at −80°C.

DNA libraries were prepared for sequencing using the homopolymer tail-mediated ligation PCR method (70). gDNA was extracted from bacterial pellets using the Qiagen DNeasy Blood and Tissue kit and sonicated with the Covaris LE220 instrument to generate 350 base pair fragments. The fragmented DNA was treated with terminal deoxytransferase to append a 3′ poly C-tail sequence, followed by two rounds of nested PCR to amplify transposon junction regions. Amplified products were multiplexed using indexing primers and sequenced on the Illumina Hi-Seq 2500 at Tufts University Core Facility.

After sequencing, reads were trimmed, quality filtered, and aligned to the ATCC *A. baumannii* 17978 genome (accession no. NZ_CP012004) using Galaxy. Each gene in the library pool was assigned a “Dval” score, calculated as the total number of reads for all transposon insertions within the gene, divided by the predicted number of reads based on gene size and total library reads. Dval scores from TPEN-treated samples were normalized to Dval scores from LB-grown samples to compute a fitness score for each gene under each condition. Fitness scores were Log2 transformed, and average Log2 fitness scores were calculated for each gene in each condition. Raw data will be deposited into Gene Expression Omnibus.

To assess the impact of ZigA on Zn-dependent fitness, we calculated a Zn-fitness *Z*-score for each gene by normalizing fitness changes within genotypes using the LB/TPEN log2-fold change (Fig. 1B; Tables S3 to S5). These *Z*-scores were then visualized in a scatter plot where the *x*-*y* coordinates were defined as the *Z*-score in WT and Δ*zigA* backgrounds. The genotype-dependent fitness score was then defined as the orthogonal distance from the *Z*-score values of a mutant to the *x* = *y* line.

For gene ontology analysis of ATCC 17978 gene products, we used the KEGG Automatic Annotation Server to query NCBI protein IDs. Entries with 100% identity were utilized to assign gene names, protein descriptions, and gene ontology terms.

### Mouse pneumonia model of infection with *A. baumannii*

To model pneumonia infection, 8-week-old female C57Bl/6 mice were purchased from Jackson Labs. Each infection group consisted of 6–10 mice. Two days before infection, WT, Δ*zigA*, Δ*sltB*, and Δ*sltB*Δ*zigA* strains were streaked onto LBA plates for single colony isolation, followed by overnight growth at 37°C with shaking at 180 rpm. On the day of infection, bacteria were subcultured into 10 mL at a 1:1,000 dilution and grown for 3.5 hours at 37°C with shaking at 180 rpm. Cells were then harvested, washed with PBS, and prepared to yield a final inoculum of 3 × 10^8^ CFU in 40 µL PBS, confirmed via serial dilutions. Inoculation was performed by intranasally pipetting 40 µL of bacterial suspension into the nasopharynx. Before infection, mice were anesthetized with an intraperitoneal injection of 2,2,2-tribromoethanol diluted in PBS. At 36 hours post-infection, mice were humanely euthanized, and CFUs were enumerated from lungs, kidneys, spleen, liver, and heart. Organ weights were measured before and after sample collection for accurate quantification of CFUs per gram of tissue.

### Transmission electron microscopy

WT, Δ*zigA*, Δ*sltB*, and Δ*sltB*Δ*zigA* cells were cultured overnight, diluted 1:50 into LB, and incubated at 37°C with shaking for 1 hour. Cells were then subcultured 1:100 into LB with or without 30 µM TPEN and grown to the mid-log phase. Fixation was performed in 2.5% glutaraldehyde in 0.1 M cacodylate buffer at room temperature for 1 hour and at 4°C for 24 hours. Fixed cells were embedded in 2% agar, equilibrated in 30% glycerol, plunge frozen in liquid ethane, and freeze substituted in 1.5% uranyl acetate at −80°C for 48 hours. Samples were raised to −30°C, washed with methanol, and infiltrated with HM20 lowicryl, which was polymerized under UV light at −30°C for 24 hours. Sections (70 nm) were cut using a Leica UC7 ultramicrotome and placed on 300 mesh copper grids. Samples were stained with 2% uranyl acetate and lead citrate. TEM imaging was conducted on a Tecnai T12 transmission electron microscope at 100 keV, with quantitative analysis performed using FIJI ROI manager with at least 100 measurements per treatment and genotype.

### Bacterial kinetic growth assays

Two days prior to experiments, all strains were streaked on LBA and grown overnight. Single colonies were then used to start overnight cultures. These cultures (WT, Δ*zigA*, Δ*sltB*, Δ*sltB*Δ*zigA*, *ΔzigA attTn7::mTn7*(amp^R^)*-zigA*, *ΔsltB attTn7::mTn7*(amp^R^)*-sltB*, and Δ*sltB*Δ*zigA attTn7::mTn7*(amp^R^)*-zigA*) were subsequently subcultured 1:1,000 into LB for 1 hour. Following this, back dilutions were inoculated at a 1:50 dilution into LB containing various experimental conditions: tetrakis-(2-pyridylmethyl)ethylenediamine (Sigma), carbenicillin (Fisher), meropenem (Patterson Veterinary Supply), sulbactam (Fisher), fosfomycin (Fisher), and vancomycin (Fisher). Growth was monitored by OD_600_ using an Epoch 2 or BioTek Synergy 2 microplate reader.

### ^70^Zn uptake assay

WT, Δ*zigA*, Δ*sltB*, and Δ*sltB*Δ*zigA* overnight cultures were back diluted into 2 mL LB, incubated for 1 hour, then subcultured into 5 mL LB ± 40 µM TPEN and grown for 3.5 hours at 37°C with shaking at 180 rpm. Optical densities were standardized across all strains, and each sample was supplemented with 25 µM ^70^ZnO, followed by a 5-minute incubation at 37°C. Cells were pelleted at 6,000 rpm for 10 minutes using a 1:1 acetone/ethanol mixture, washed twice with PBS, and transferred to metal-free 15 mL conical tubes. The cells were digested overnight in 50% Optima-grade nitric acid at 65°C and then diluted with UltraPure water for analysis by ICP-MS. Elemental quantification was conducted using an Agilent 7700 ICP-MS attached to an ASX-560 autosampler. The settings for analysis were cell entrance = −40 V, cell exit = −60 V, plate bias = −60 V, OctP bias = −18 V, and helium flow = 4.5 mL/min. Optimal voltages for extract 2, omega bias, omega lens, OctP RF, and deflect were empirically determined. Calibration curves for elements were generated using ARISTAR ICP standard mix. Samples were introduced by a peristaltic pump with 0.5-mm-internal-diameter tubing through a MicroMist borosilicate glass nebulizer. They were initially taken up at 0.5 rps for 30 seconds, followed by 30 seconds at 0.1 rps to stabilize the signal. Spectrum mode analysis was performed at 0.1 rps, collecting three points across each peak and conducting three replicates of 100 sweeps for each element. The sampling probe and tubing were rinsed with 2% nitric acid for 20 seconds at 0.5 rps between each sample. Data were acquired and analyzed using Agilent MassHunter workstation software version A.01.02, with ^70^Zn values normalized to total cellular sulfur (^34^S).

### Ethidium bromide uptake

WT, Δ*zigA*, Δ*sltB*, and Δ*sltB*Δ*zigA* cells were grown overnight at 37°C with shaking at 180 rpm and then back diluted at 1:1,000 in 2 mL LB for 1 hour. The cells were then subcultured into LB ± 40 µM TPEN, normalized to an OD_600_ of 0.3, and washed once with PBS. Cell suspensions were transferred to black 96-well plates and supplemented with ethidium bromide (1 µg/mL, Sigma). Fluorescence was immediately measured at 20-second intervals (ex: 530 nm; em: 600 nm) using a BioTek Cytation 5 imaging reader. Data represent results from at least three independent experiments.

### Statistical analysis and quantification

Raw data were initially recorded in Microsoft Excel, imported into GraphPad Prism for statistical analysis, and visualized using Envision Canvas software. Data were analyzed using one- or two-way ANOVA as specified in the figure legends. Asterisks denote statistical significance (*P < 0.05, **P < 0.01, ***P < 0.001, and ****P < 0.0001), and significant values are indicated in each figure. Details regarding specific statistical tests, significance levels, data dispersion, group sizes, and measurement precision are provided in the figure legends.

## SUPPLEMENTAL MATERIAL

Supplemental figures iai.00422-24-s0001.docx Fig. S1 to S6.

Supplemental tables iai.00422-24-s0002.docx Tables S1 to S4.

Table S5 iai.00422-24-s0003.xlsx Relevant hits to the Tn-seq screen performed in this study.

ASM does not own the copyrights to Supplemental Material that may be linked to, or accessed through, an article. The authors have granted ASM a non-exclusive, world-wide license to publish the Supplemental Material files. Please contact the corresponding author directly for reuse.
