# Supplementary material for: The interplay between Acinetobacter baumannii ZigA and SltB promotes zinc homeostasis and cell envelope integrity
Source: Infect Immun. 2025 Jan 23;93(2):e00422-24. doi: 10.1128/iai.00422-24 (PMC11834433; doi:10.1128/iai.00422-24)
Supplement: Supplemental figures — Fig. S1 to S6. [file iai.00422-24-s0001.docx]

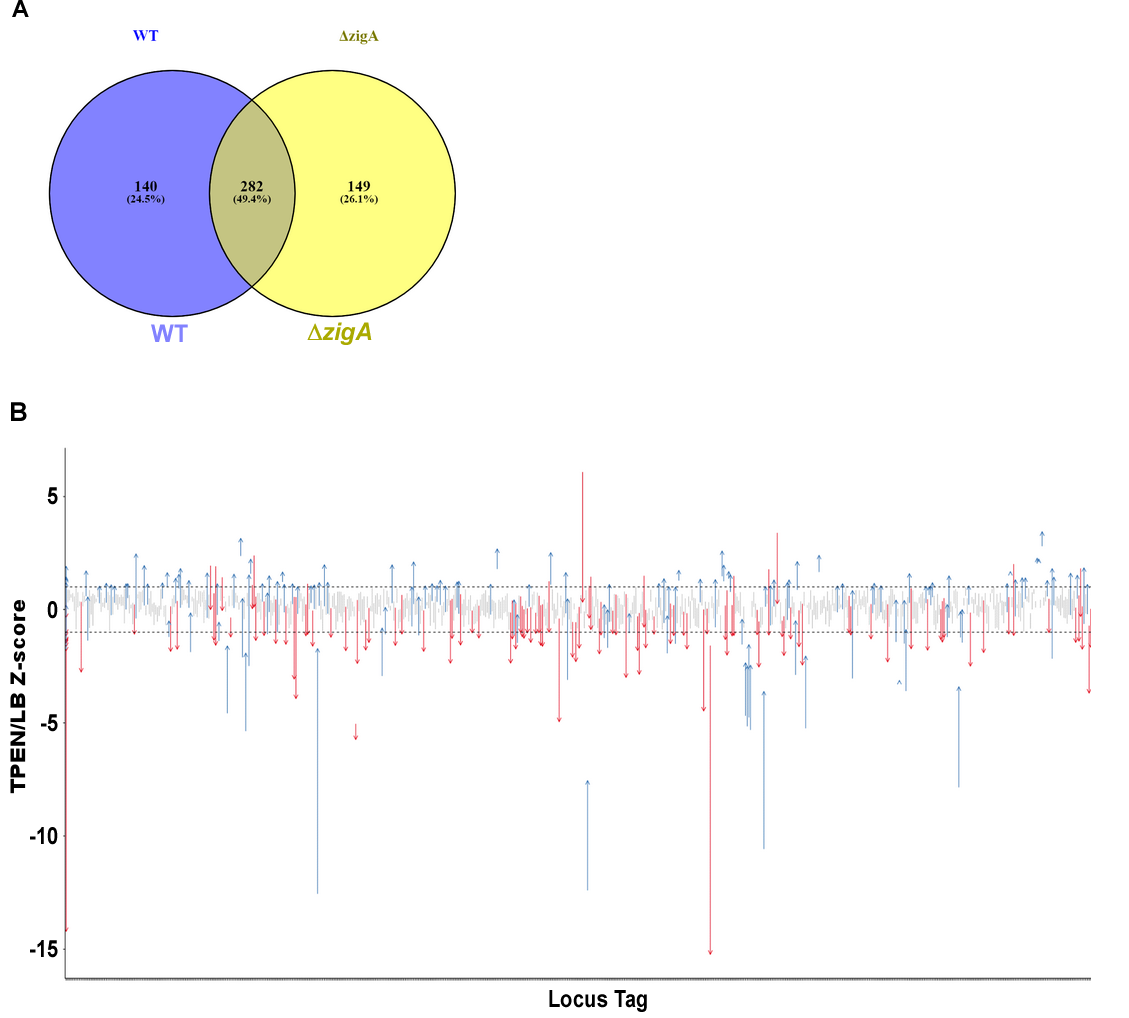


**Supplementary Figure 1: ZigA supports essential cellular processes. (A)** A Venn diagram indicating the number of essential and conditionally essential genes in the WT and Δ*zigA* transposon libraries. Essentiality was determined by quantifying genes in both libraries with less than 50 total reads. **(B)** Fitness profiles across all conditions were analyzed to identify new gene relationships with *zigA*. Data are represented as a Z-score of gene fitness in the Δ*zigA* library divided by WT. The data are then further separated by genomic position to show fitness similarities due to genomic location. Genes ascribed as blue are genes whose fitness score increased in the Δ*zigA* library background and genes ascribed as red have a fitness score that decreased in the Δ*zigA* library. Dotted lines indicate a Z-score ± 1.5.


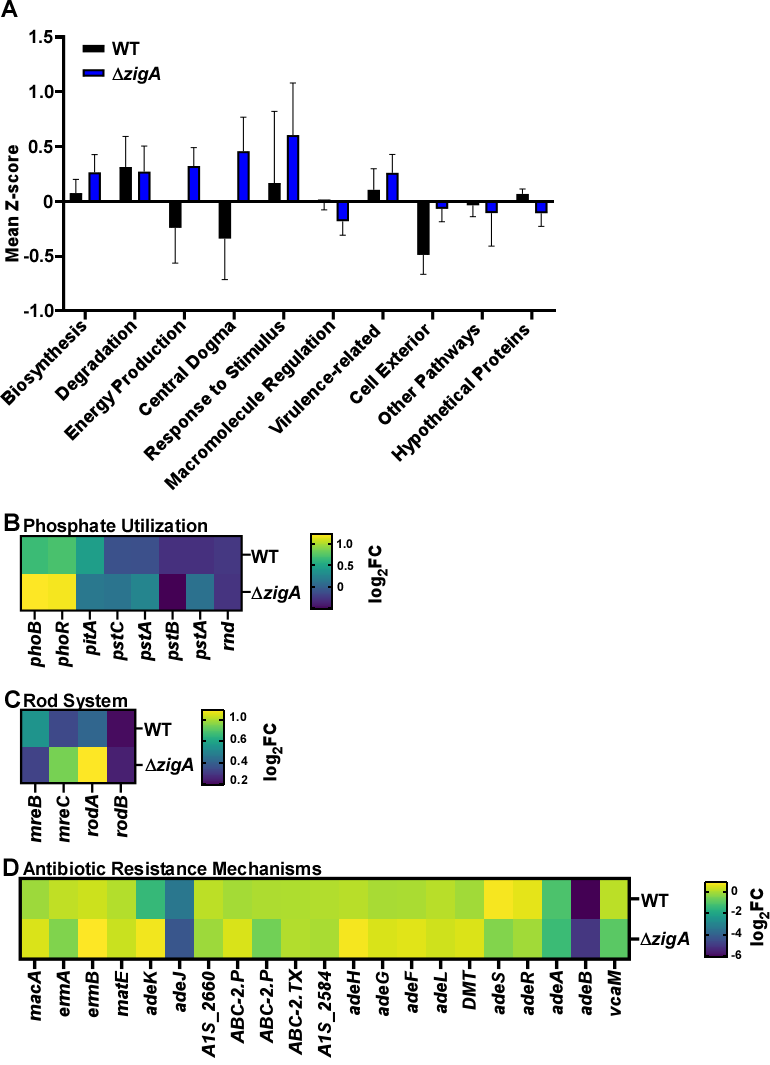


**Supplementary Figure 2: ZigA influences many critical cellular pathways during zinc starvation.** **(A)** A gene ontology (GO)-term enrichment analysis of genes in functional categories indicated as a Z-score for either the WT or Δ*zigA* libraries. Heatmaps indicating the fitness change of genes in functional categories related to **(B)** phosphate utilization, **(C)** the rod system, and **(D)** antibiotic resistance.

**
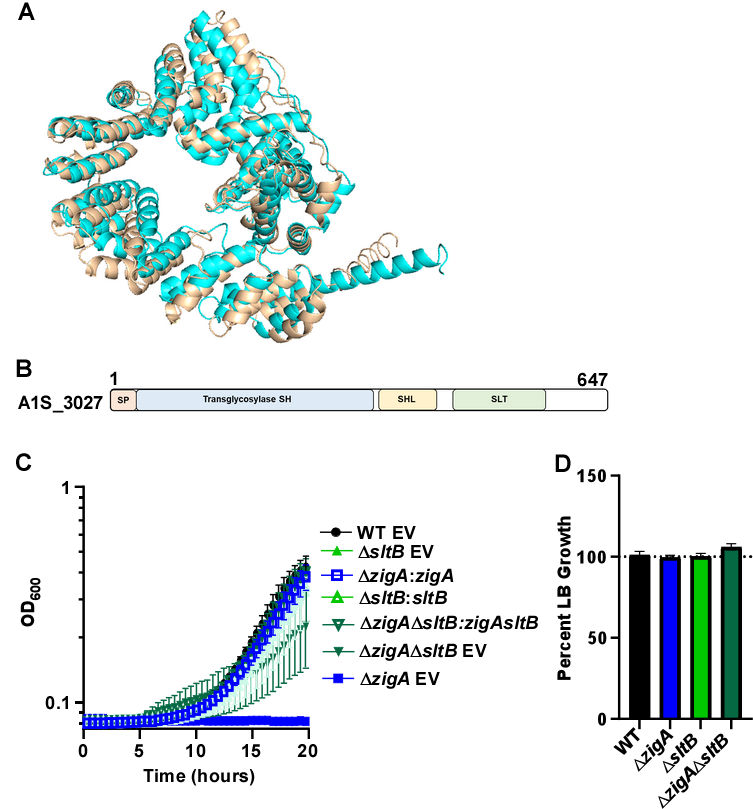
**

**Supplementary Figure 3: Combined inactivation of *zigA* and *sltB* which encodes a predicted soluble lytic transglycosylase, impacts the ability of *Acinetobacter baumannii* to thrive in low Zn environments.** **(A)** A predictive AlphaFold structure of *A. baumannii* A1S_3027 (SltB) (light blue) is shown overlaid to the crystal structure of *Pseudomonas aeruginosa* Slt (wheat). **(B)** Schematic indicating the conserved Slt structures that A1S_3027 (SltB) is predicted to contain, including a signaling peptide (SP), a transglycosylase super helical domain (SH), a super helical linker (SHL), and a soluble lytic transglycosylase domain (SLT). **(C)** WT, Δ*zigA*, Δ*sltB*, and Δ*zigA*Δ*sltB* integration controls and complementation constructs (Δ*zigA attTn7::mTn7* (amp^R^)-*zigA*, Δ*sltB attTn7::mTn7*(amp^R^)-*sltB*, Δ*sltB*Δ*zigA attTn7::mTn7*(amp^R^)-*sltB-zigA*) were grown ± 60 μM TPEN with OD_600_ monitored over time. **(D)** Percentage of rich media (LB) growth as determined by OD_600_ at 16 hours after growth in either 60 μM TPEN or 60 μM TPEN ± 60 μM ZnCl_2_ compared with untreated strains.


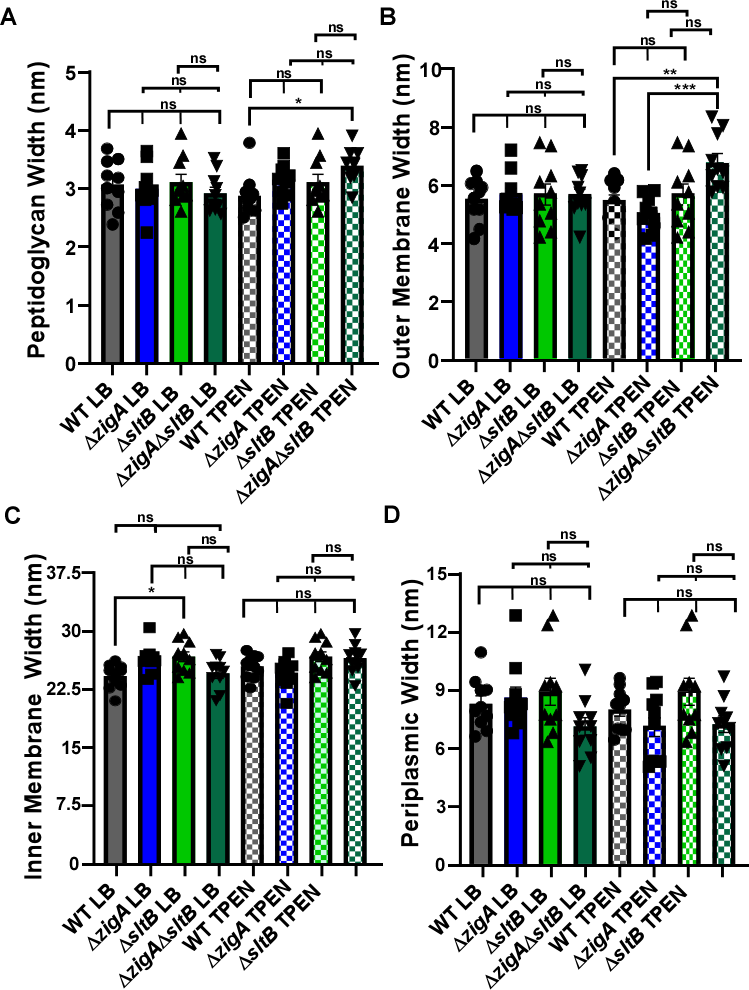


**Supplementary Figure 4: ZigA-SltB functional interactions alter peptidoglycan and outer membrane width structures during Zn starvation.** **(A)** Assessments of electron micrographs of WT, Δ*zigA*, Δ*sltB*, and Δ*zigA*Δ*sltB* in LB ± 40 μM TPEN. Each measurement is represented as an average of 10 measurements per cell, with 10 cells represented per genotype. **(A)** Peptidoglycan, **(B)** outer membrane, **(C)** inner membrane, and **(D)** periplasmic widths of cells were assessed using ImageJ software. *p<0.05,**p<0.01, ***p<0.001 as determined by One-way ANOVA with Šídák's multiple comparisons test

**
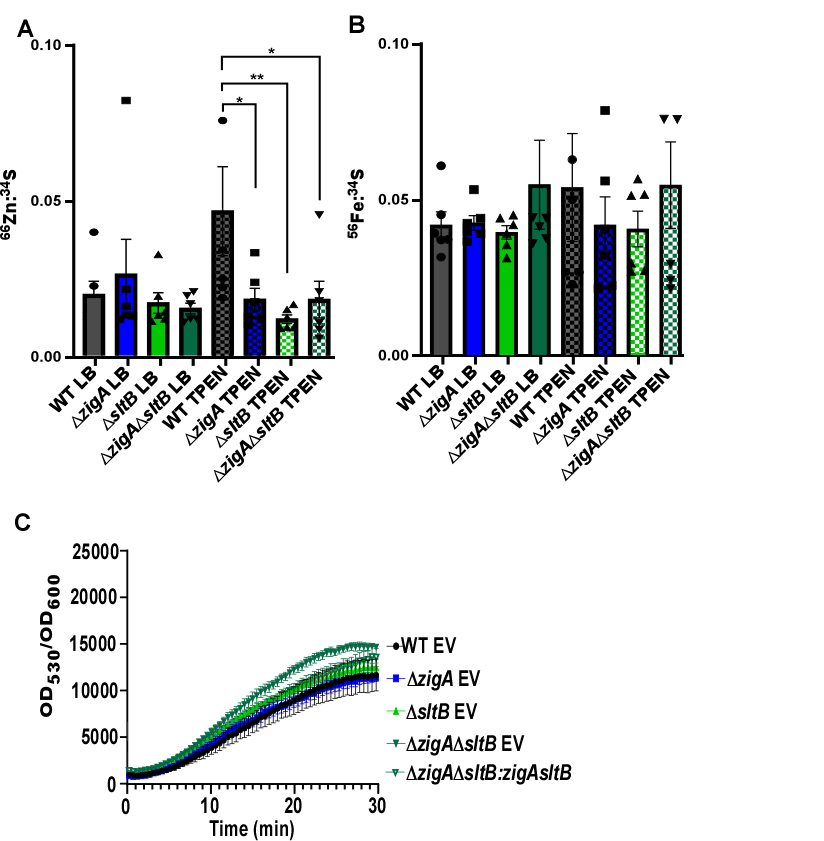
**

**Supplementary Figure 5: ZigA-SltB functional interactions alter cell envelope integrity.** **(A)** ^66^Zn, and **(B)** ^56^Fe uptake were quantified by ICP-MS and normalized to ^34^S for WT, Δ*zigA*, Δ*sltB*, and Δ*zigA*Δ*sltB* ± 40 μM TPEN or 32 μg/mL vancomycin. *p<0.05 and **p<0.01 by One-way ANOVA. **(C)** Ethidium bromide uptake following early stationary growth in 40 μM TPEN.


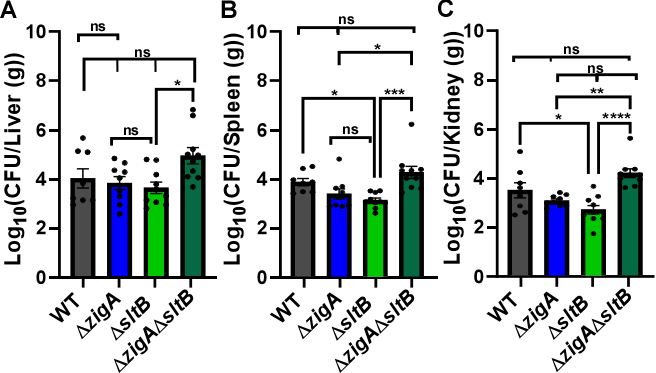


**Supplementary Figure 6: ZigA-SltB functional interactions alter *A. baumannii* infectivity.** **(A-C)** WT, Δ*zigA*, Δ*sltB*, and Δ*zigA*Δ*sltB* bacterial burdens removed from the **(A)** liver, **(B)** spleen, or **(C)** kidney at 36 hpi. *p<0.05, **p<0.01, ***p<0.001, ****p<0.0001 as determined by one-way ANOVA with Tukey multiple comparisons test (n=8-10 biological replicates per genotype).
