# Supplementary material for: The interplay between Acinetobacter baumannii ZigA and SltB promotes zinc homeostasis and cell envelope integrity
Source: Infect Immun. 2025 Jan 23;93(2):e00422-24. doi: 10.1128/iai.00422-24 (PMC11834433; doi:10.1128/iai.00422-24)
Supplement: Supplemental tables — Tables S1 to S4. [file iai.00422-24-s0002.docx]

**Table S1: Strains used in this study.**

| **Strain** | **Characteristics** | **Source** |
| --- | --- | --- |
| *Acinetobacter baumannii*  ATCC 17978VU | Wildtype | ATCC (Wijers *et al*., 2021) |
| *Escherichia coli* DH5α |  | Lab stock |
| *Escherichia coli* SM10λ*pir* pJNW684 | Contains Himar1 transposon | Alan Hauser (Wang *et al*., 2014) |
| *Escherichia coli* DH5α pFLp2 | *A. baumannii* allelic exchange vector with *sacB* (pFLp2) | Herbert Schweizer  (Hoang *et al*., 1998) |
| *Escherichia coli* pUC18-K1 | Plasmid containing *aphA* gene | (Ménard *et al.*, 1993) |
| *Escherichia coli* DH5α pFLp2-Δ*zigA* | *zigA* knockout vector | This study |
| Acinetobacter baumannii ATCC 17978VU Δ*zigA* | Δ*zigA* | This study |
| *Escherichia coli* DH5α pFLp2-Δ*sltB::aphA* | *sltB* knockout vector | This study |
| *Acinetobacter baumannii* ATCC 17978VU Δ*sltB::aphA* | Δ*sltB::aphA* | This study |
| *Acinetobacter baumannii* ATCC 17978VU Δ*zigA*Δ*sltB* | Δ*zigA*Δ*sltB::aphA* | This study |
| *Escherichia coli* HB101 pRK2013 | Helper plasmid containing RK2 transfer genes | (Figurski and Helinski, 1979) |
| *Escherichia coli* pTNS2 | Transposase expression | (Choi *et al.*, 2005) |
| *Escherichia coli* pKNOCK-mTn7(amp^R^) | mini-Tn7-Amp | (Carruthers *et al*., 2013) |
| *Acinetobacter baumannii* ATCC 17978VU *attTn7:: mTn7*(amp^R^) | WT empty mTn7 integration control | This study |
| *Acinetobacter baumannii* ATCC 17978VU Δ*zigA* *attTn7::mTn7*(amp^R^) | Δ*zigA* empty mTn7 integration control | This study |
| *Acinetobacter baumannii* ATCC 17978VU Δ*sltB::aphA* *attTn7::mTn7*(amp^R^) | Δ*sltB::aphA* empty mTn7 integration control | This study |
| *Acinetobacter baumannii* ATCC 17978VU Δ*zigA*Δ*sltB::aphA* *attTn7::mTn7*(amp^R^) | Δ*zigA*Δ*sltB::aphA* empty mTn7 integration control | This study |
| *Acinetobacter baumannii* ATCC 17978VU *attTn7::mTn7*(amp^R^)*-zigA* | *zigA* complementation strain | This study |
| *Escherichia coli* DH5αλpir pKNOCK-mTn7(amp^R^)-*zigA* | *zigA* complementation vector | This study |
| *Acinetobacter baumannii* ATCC 17978VU *attTn7:: mTn7*(amp^R^)-*sltB* | *sltB* complementation strain | This study |
| *Escherichia coli* DH5αλpir pKNOCK-mTn7(amp^R^)-*sltB* | *sltB* complementation vector | This study |
| *Acinetobacter baumannii* ATCC 17978VU *attTn7::mTn7*(amp^R^)*-zigA-sltB* | *zigA* and *sltB* complementation strain | This study |
| *Escherichia coli* DH5αλpir pKNOCK-mTn7(amp^R^*)zigA*-*sltB* | *zigA* and *sltB* complementation vector | This study |

**Table S2: Oligonucleotides used in this study.**

| **Primer Name** | **5’-3’ Sequence** | **Description** |
| --- | --- | --- |
| **Tn-seq** |  |  |
| LP28 | AAGCAGAAGACGGCATACG | PCR 1 Tn-seq 5’ primer |
| olj376 | GTGACTGGAGTTCAGACGTGTGCTCTTCCGATCTGGGG  GGGGGGGGGGGG | PCR 1 Tn-seq 3’ primer |
| AC105 | AATGATACGGCGACCACCGAGATCTACACTCTTTGACCG  GGGACTTATCATCCAACCTGTTA | PCR 2 Tn-seq 5’ |
| ERG135 | GACCGGGGACTTATCATCCAACCTGTTA | Tn-specific Illumina sequencing primer |
| BC43 | CAAGCAGAAGACGGCATACGAGATGTAGCCGTGACTGG  AGTTCAGACGTGTGCTCTTCCGATCT | PCR 2’ Tn-seq 3’ primer with custom 6bp barcode |
| BC44 | CAAGCAGAAGACGGCATACGAGATTACAAGGTGACTGG  AGTTCAGACGTGTGCTCTTCCGATCT | PCR 2’ Tn-seq 3’ primer with custom 6bp barcode |
| BC45 | CAAGCAGAAGACGGCATACGAGATTGTTGACTGTGACT  GGAGTTCAGACGTGTGCTCTTCCGATCT | PCR 2’ Tn-seq 3’ primer with custom 8bp barcode |
| BC46 | CAAGCAGAAGACGGCATACGAGATACGGAACTGTGACT  GGAGTTCAGACGTGTGCTCTTCCGATCT | PCR 2’ Tn-seq 3’ primer with custom 8bp barcode |
| BC47 | CAAGCAGAAGACGGCATACGAGATTCTGACATGTGACT  GGAGTTCAGACGTGTGCTCTTCCGATCT | PCR 2’ Tn-seq 3’ primer with custom 8bp barcode |
| BC48 | CAAGCAGAAGACGGCATACGAGATCGGGACGGGTGACT  GGAGTTCAGACGTGTGCTCTTCCGATCT | PCR 2’ Tn-seq 3’ primer with custom 8bp barcode |
| BC49 | CAAGCAGAAGACGGCATACGAGATGTGCGGACGTGACT  GGAGTTCAGACGTGTGCTCTTCCGATCT | PCR 2’ Tn-seq 3’ primer with custom 8bp barcode |
| BC50 | CAAGCAGAAGACGGCATACGAGATCGTTTCACGTGACT  GGAGTTCAGACGTGTGCTCTTCCGATCT | PCR 2’ Tn-seq 3’ primer with custom 8bp barcode |
| BC51 | CAAGCAGAAGACGGCATACGAGATAAGGCCACGTGACT  GGAGTTCAGACGTGTGCTCTTCCGATCT | PCR 2’ Tn-seq 3’ primer with custom 8bp barcode |
| BC52 | CAAGCAGAAGACGGCATACGAGATACCGAAACGTGACT  GGAGTTCAGACGTGTGCTCTTCCGATCT | PCR 2’ Tn-seq 3’ primer with custom 8bp barcode |
| BC53 | CAAGCAGAAGACGGCATACGAGATTACGTACGGTGACT  GGAGTTCAGACGTGTGCTCTTCCGATCT | PCR 2’ Tn-seq 3’ primer with custom 8bp barcode |
| BC54 | CAAGCAGAAGACGGCATACGAGATATCCACTCGTGACT  GGAGTTCAGACGTGTGCTCTTCCGATCT | PCR 2’ Tn-seq 3’ primer with custom 8bp barcode |
| BC55 | CAAGCAGAAGACGGCATACGAGATATATCAGTGTGACT  GGAGTTCAGACGTGTGCTCTTCCGATCT | PCR 2’ Tn-seq 3’ primer with custom 8bp barcode |
| BC56 | CAAGCAGAAGACGGCATACGAGATAAAGGAATGTGACT  GGAGTTCAGACGTGTGCTCTTCCGATCT | PCR 2’ Tn-seq 3’ primer with custom 8bp barcode |
| ***zigA* and *sltB* mutant construction and confirmation** |  |  |
| *zigA*_Up_F | GGTTAAAAAGGATCGATCCTCTAGAC  CGATATGTAAACAGATCTTAG | pFLP2 cloning, 5’ flanking region, Forward for *zigA* KO construct |
| *zigA*_Up_R | GGCTTTTTTAGGCTGGCCATTTAAATATG | pFLP2 cloning, 5’ flanking region, Reverse for *zigA* KO construct |
| *zigA*_Down_F | ATGGCCAGCCTAAAAAAGCCCCTATTTTCAAG | pFLP2 cloning, 3’ flanking region, Forward for *zigA* KO construct |
| *zigA*_Down_R | AAGTTCCTATTCTCTAGGGGGATCCACGGAAATTATGG  CGTCAC | pFLP2 cloning, 3’ flanking region, Reverse for *zigA* KO construct |
| *sltB*_Kan_F | GAGTCGTGTGTGACTAACTAGGAGGAATAAATGG | 5’ primer for amplifying *aphA* for *sltB* KO construct |
| *sltB*_Kan_R | GCGTTGGAATTCATTATTCCCTCCAGGTAC | 3’ primer for amplifying *aphA* for *sltB* KO construct |
| *sltB*_Up_F | GGTTAAAAAGGATCGATCCTCTAGACATTTTGACCAAG  TAAGCTG | pFLP2 cloning, 5’ flanking region, Forward for *sltB* KO construct |
| *sltB*_Up_R | TAGTTAGTCACACACGACTCTTGTTTGATG | pFLP2 cloning, 5’ flanking region, Reverse for *sltB* KO construct |
| *sltB*_Down_F | GGAATAATGAATTCCAACGCGATCATCAC | pFLP2 cloning, 3’ flanking region, Forward for *sltB* KO construct |
| *sltB*_Down_R | AAGTTCCTATTCTCTAGGGGGATCCACGGAATAATAAG  CTGTAACC | pFLP2 cloning, 3’ flanking region, Reverse for *sltB* KO construct |
| ***zigA* and *sltB* complementation** |  |  |
| *sltB*_Comp_F | CATGCATGAGCTCACTAGTGGATCCTGACACCCTTGAGTTTC | Cloning into pKNOCK, forward |
| *sltB*_Comp_R | GCAAGGCCTTCGCGAGGTACCTAAGGTGATGATCGCGTTG | Cloning into pKNOCK, reverse |
| *zigA*_Comp_F | CATGCATGAGCTCACTAGTGGATCCAAGCAATTTTTTCAAACACTAAG | Cloning into pKNOCK, forward |
| *zigA*_Comp_R | GCAAGGCCTTCGCGAGGTACCTTAAGCGATCAACATCGTTTC | Cloning into pKNOCK, reverse |
| *zigAsltB*_Comp_F  *zigA* | CATGCATGAGCTCACTAGTGGATCCAAGCAATTTTTTCAAACACTAAG | Cloning into pKNOCK, forward |
| *zigAsltB*_Comp_R  *zigA* | AGGGTGTCAGTTAAGCGATCAACATCGTTTC | Cloning into pKNOCK, reverse |
| *zigAsltB*_Comp_F  *sltB* | GATCGCTTAACTGACACCCTTGAGTTTC | Cloning into pKNOCK, forward |
| *zigAsltB*_Comp_R  *sltB* | GCAAGGCCTTCGCGAGGTACCTAAGGTGATGATCGCGTTG | Cloning into pKNOCK, reverse |

**Table S3:** Genes with altered fitness in the WT library during Zn Depletion (Complete analysis can be found in Table S5)

| **Locus Tag (ACX60_)** | **Locus Tag (A1S_)** | | **Gene name** | **AVG LB** | **AVG TPEN** | **AVG (TPEN/LB)** | **P-value TPEN/LB** |
| --- | --- | --- | --- | --- | --- | --- | --- |
| *ACX60_14465* | | *A1S_0780* | *mrp, NUBPL* | -2.574483354 | -7.40429 | 2.876029111 | 0.000007 |
| *ACX60_05430* | | *A1S_2427* | *ampG* | -2.255121226 | -6.34623 | 2.814142381 | 0.000184 |
| *ACX60_17275* | | *A1S_0162* | *-* | -2.144631404 | -5.75938 | 2.685487136 | 0.03398 |
| *ACX60_09130* | | *A1S_1750* | *adeB* | -0.682201128 | -5.48604 | 8.041673013 | <0.000001 |
| *ACX60_05950* | | *A1S_2317* | *rlpA* | -2.590034851 | -5.48478 | 2.117645349 | 0.026767 |
| *ACX60_10835* | | *A1S_1462* | *-* | -1.957058449 | -5.42685 | 2.77296397 | 0.000002 |
| *ACX60_16050* | | *A1S_0414* | *nudH* | -3.273583592 | -5.23151 | 1.598098272 | 0.028906 |
| *ACX60_17775* | | *A1S_0059* | *gtr8* | -3.694263036 | -4.89765 | 1.325745848 | 0.153382 |
| *ACX60_03830* | | *A1S_2736* | *adeJ* | -2.087941234 | -4.76505 | 2.282175919 | <0.000001 |
| *ACX60_15075* | | *A1S_0610* | *proC* | -3.682297009 | -4.69259 | 1.27436544 | 0.485699 |
| *ACX60_15640* | | *A1S_0493* | *prc* | -2.571527223 | -4.66979 | 1.815959872 | 0.000002 |
| *ACX60_15955* | | *A1S_0430* | *lpsB* | -1.577575645 | -4.58414 | 2.905811702 | 0.000178 |
| *ACX60_15950* | | *A1S_0431* | *lpxL, htrB* | -2.788478655 | -4.54255 | 1.62904403 | 0.000006 |
| *ACX60_03040* | | *A1S_2894* | *aspS* | -4.02454266 | -4.5406 | 1.128228566 | 0.314811 |
| *ACX60_04600* | | *A1S_2592* | *tolR* | -2.475268857 | -4.50266 | 1.819059373 | 0.000032 |
| *ACX60_04855* | | *A1S_2543* | *-* | -1.976086487 | -4.42287 | 2.238199004 | 0.101003 |
| *ACX60_01995* | | *A1S_3100* | *mlaC* | -1.369795924 | -4.15399 | 3.032559206 | 0.243474 |
| *ACX60_09670* | | *-* | *-* | -2.28097628 | -4.05329 | 1.777000041 | 0.031466 |
| *ACX60_00870* | | *A1S_3316* | *prfB* | -4.111288202 | -4.01204 | 0.975859228 | 0.42147 |
| *ACX60_13550* | | *A1S_3583* | *-* | -2.852890969 | -3.94985 | 1.384509328 | 0.335853 |
| *ACX60_03265* | | *A1S_2846* | *cysI* | -4.548933246 | -3.88357 | 0.853732659 | 0.795614 |
| *ACX60_01985* | | *A1S_3102* | *mlaE, linK* | -0.970553953 | -3.87537 | 3.992943638 | 0.512226 |
| *ACX60_09735* | | *A1S_1644* | *-* | -3.477831528 | -3.82151 | 1.098819467 | 0.944117 |
| *ACX60_10355* | | *A1S_1548* | *amgK* | -3.230101804 | -3.75553 | 1.162667144 | 0.968551 |
| *ACX60_02025* | | *A1S_3095* | *recG* | -3.734897898 | -3.67869 | 0.984951737 | 0.910996 |
| *ACX60_10460* | | *A1S_1528* | *putA* | -2.756409584 | -3.64456 | 1.322213012 | 0.000869 |
| *ACX60_11430* | | *A1S_1347* | *paaX* | -3.159348754 | -3.62878 | 1.148583944 | 0.467795 |
| *ACX60_13375* | | *A1S_1001* | *cysN* | -3.640379055 | -3.58029 | 0.98349328 | 0.733316 |
| *ACX60_01215* | | *A1S_3254* | *kup* | -4.033268077 | -3.56895 | 0.884877169 | 0.626793 |
| *ACX60_11235* | | *A1S_3659* | *-* | -1.662005306 | -3.55674 | 2.140031132 | 0.248441 |
| *ACX60_05175* | | *A1S_2477* | *IDH1, IDH2, icd* | -4.792439567 | -3.50364 | 0.731076793 | 0.000176 |
| *ACX60_11190* | | *A1S_3662* | *-* | -3.048513716 | -3.44984 | 1.131645187 | 0.098493 |
| *ACX60_03825* | | *A1S_2737* | *adeK* | -2.225512297 | -3.43853 | 1.545051633 | 0.000118 |
| *ACX60_17365* | | *A1S_0143* | *znuB* | -1.084355457 | -3.43011 | 3.163266756 | 0.036922 |
| *ACX60_02280* | | *A1S_3064* | *RP-S5, MRPS5, rpsE* | -3.164576509 | -3.42514 | 1.082337342 | 0.187073 |
| *ACX60_15920* | | *A1S_0438* | *-* | -0.872262694 | -3.3556 | 3.847009499 | 0.166164 |
| *ACX60_11390* | | *A1S_1351* | *katE, CAT, catB, srpA* | -2.862915349 | -3.33278 | 1.164119442 | 0.422733 |
| *ACX60_03270* | | *A1S_2845* | *-* | -2.757938841 | -3.33118 | 1.207852998 | 0.562815 |
| *ACX60_17350* | | *A1S_0146* | *znuA* | -1.381969511 | -3.32617 | 2.406832103 | 0.000151 |
| *ACX60_16895* | | *A1S_0238* | *thrC* | -4.133250837 | -3.31743 | 0.802621108 | 0.690904 |
| *ACX60_03885* | | *A1S_2697* | *cysG* | -3.080819685 | -3.28121 | 1.06504514 | 0.000066 |
| *ACX60_12880* | | *-* | *-* | -2.379344448 | -3.25602 | 1.36845098 | 0.004171 |
| *ACX60_06475* | | *A1S_3796* | *-* | -3.098642664 | -3.24723 | 1.047951053 | 0.973323 |
| *ACX60_10830* | | *A1S_1463* | *cysE* | -2.515313873 | -3.22679 | 1.28285873 | 0.015726 |
| *ACX60_00760* | | *A1S_3337* | *gshB* | -1.925575633 | -3.16895 | 1.645713134 | 0.102985 |
| *ACX60_05085* | | *A1S_2497* | *proB* | -2.450151608 | -3.15943 | 1.289483821 | 0.00003 |
| *ACX60_01990* | | *A1S_3101* | *mlaD, linM* | -0.066175483 | -3.12299 | 47.19249893 | 0.019292 |
| *ACX60_17755* | | *A1S_3484* | *ugd* | -2.991443144 | -3.10089 | 1.036586505 | 0.005159 |
| *ACX60_09565* | | *A1S_1674* | *-* | -2.801682974 | -3.09345 | 1.104138444 | 0.294874 |
| *ACX60_11555* | | *A1S_1320* | *soxR* | -1.957221332 | -3.09187 | 1.579725526 | 0.053248 |
| *ACX60_18090* | | *A1S_2963* | *purK* | -3.130348827 | -3.02703 | 0.966993942 | 0.00346 |
| *ACX60_04595* | | *A1S_2593* | *tolA* | -1.243682366 | -3.01595 | 2.425015635 | 0.82502 |
| *ACX60_09465* | | *A1S_1688* | *K09902* | -1.842176485 | -3.01299 | 1.635557448 | 0.04408 |
| *ACX60_13855* | | *A1S_0906* | *hemB, ALAD* | -3.115356412 | -2.97109 | 0.953693041 | 0.035817 |
| *ACX60_00290* | | *A1S_3425* | *purC* | -2.096024831 | -2.96777 | 1.415904309 | 0.00225 |
| *ACX60_06490* | | *A1S_2216* | *-* | -2.975333827 | -2.96302 | 0.995861727 | 0.502773 |
| *ACX60_05910* | | *A1S_2324* | *metap* | -3.275668988 | -2.92195 | 0.892015868 | 0.000795 |
| *ACX60_01970* | | *A1S_3104* | *-* | -2.397654569 | -2.89488 | 1.207379143 | 0.043059 |
| *ACX60_17170* | | *A1S_0181* | *-* | -2.770941688 | -2.86863 | 1.035255402 | 0.301693 |
| *ACX60_10340* | | *A1S_1551* | *parA, soj* | -3.272405199 | -2.86022 | 0.874042633 | 0.000018 |
| *ACX60_14705* | | *A1S_0737* | *metE* | -3.351164507 | -2.84628 | 0.849341192 | 0.462554 |
| *ACX60_01980* | | *A1S_3103* | *mlaF, linL, mkl* | -0.530214236 | -2.81353 | 5.306406332 | 0.44136 |
| *ACX60_05610* | | *A1S_2388* | *ABC.FEV.P* | -0.919498213 | -2.7843 | 3.02806334 | 0.000742 |
| *ACX60_13230* | | *A1S_1030* | *lon protease* | -4.19631718 | -2.77261 | 0.66072406 | 0.000202 |
| *ACX60_12040* | | *A1S_3632* | *-* | -2.492861031 | -2.75537 | 1.10530302 | 0.065785 |
| *ACX60_17355* | | *A1S_0145* | *zur* | -2.428198458 | -2.73052 | 1.124503453 | 0.003039 |
| *ACX60_00740* | | *A1S_3340* | *pyrE* | -2.713802071 | -2.72197 | 1.00300854 | 0.175221 |
| *ACX60_03340* | | *A1S_2832* | *ppiC* | -1.422477724 | -2.7102 | 1.905264423 | 0.382072 |
| *ACX60_10350* | | *A1S_1549* | *murU* | -3.122428993 | -2.70181 | 0.865290956 | 0.000074 |
| *ACX60_08710* | | *A1S_1820* | *-* | -2.052177462 | -2.70029 | 1.315817517 | 0.038744 |
| *ACX60_14040* | | *A1S_0869* | *tuf, TUFM* | -2.831759682 | -2.68662 | 0.948747201 | 0.650227 |
| *ACX60_03705* | | *A1S_2761* | *prpF* | -4.326884044 | -2.68484 | 0.62050159 | 0.003289 |
| *ACX60_16700* | | *A1S_0279* | *tuf, TUFM* | -2.658924445 | -2.6288 | 0.988669349 | 0.316653 |
| *ACX60_09605* | | *-* | *-* | 0.083880157 | -2.6216 | -31.25415417 | 0.000011 |
| *ACX60_05605* | | *A1S_2389* | *ABC.FEV.P* | -0.523661052 | -2.60054 | 4.966069909 | 0.475127 |
| *ACX60_07595* | | *-* | *-* | -2.233259355 | -2.56329 | 1.147778532 | 0.017104 |
| *ACX60_12825* | | *A1S_1105* | *-* | -2.782759163 | -2.55139 | 0.916856945 | 0.894817 |
| *ACX60_12610* | | *-* | *-* | -1.925131765 | -2.54233 | 1.320598749 | 0.126705 |
| *ACX60_13740* | | *A1S_0928* | *betI* | -2.788179384 | -2.50978 | 0.900151793 | 0.568926 |
| *ACX60_17990* | | *A1S_0018* | *slpA* | -1.25889868 | -2.50715 | 1.991539548 | 0.023541 |
| *ACX60_15985* | | *A1S_0424* | *E3.5.1.1, ansA, ansB* | -3.009259899 | -2.50575 | 0.832680526 | 0.287292 |
| *ACX60_05620* | | *A1S_2386* | *ABC.FEV.S* | -0.469228669 | -2.48554 | 5.297074076 | 0.000001 |
| *ACX60_12660* | | *A1S_1140* | *rnhB* | -2.095738855 | -2.48168 | 1.184154083 | 0.006232 |
| *ACX60_10825* | | *A1S_1464* | *trmJ* | -2.126084327 | -2.47392 | 1.163606116 | 0.004488 |
| *ACX60_04970* | | *A1S_2518* | *era, ERAL1* | -2.52623982 | -2.45497 | 0.971789823 | 0.000031 |
| *ACX60_04400* | | *A1S_2632* | *lysA* | -3.07595731 | -2.45368 | 0.797694838 | 0.000241 |
| *ACX60_02910* | | *A1S_2912* | *tgt, QTRT1* | -2.317677951 | -2.4418 | 1.053554509 | 0.022495 |
| *ACX60_15500* | | *A1S_0523* | *-* | -3.751235248 | -2.40665 | 0.641560721 | 0.024934 |
| *ACX60_15735* | | *A1S_0473* | *K07336* | -3.273869415 | -2.39681 | 0.732103679 | 0.033127 |
| *ACX60_09260* | | *A1S_1727* | *hypT, qseD* | -3.774218587 | -2.39257 | 0.633923667 | 0.27125 |
| *ACX60_10160* | | *A1S_3686* | *-* | -1.950335503 | -2.38494 | 1.222835051 | 0.073053 |
| *ACX60_07150* | | *-* | *-* | -2.187505627 | -2.37029 | 1.083560345 | 0.000642 |
| *ACX60_09650* | | *A1S_1659* | *cobP, cobU* | -2.036971159 | -2.36821 | 1.162613587 | 0.497501 |
| *ACX60_15660* | | *A1S_0489* | *proA* | -1.543629733 | -2.36564 | 1.532516271 | 0.000145 |
| *ACX60_14885* | | *A1S_0701* | *-* | -2.677240465 | -2.35346 | 0.879063588 | 0.000057 |
| *ACX60_15615* | | *A1S_0499* | *rlmN* | -2.92633335 | -2.33832 | 0.799061124 | 0.001652 |
| *ACX60_09615* | | *A1S_3712* | *-* | -0.220905752 | -2.33106 | 10.55230163 | 0.016168 |
| *ACX60_09515* | | *A1S_1681* | *-* | -2.485653506 | -2.31431 | 0.931067928 | 0.002211 |
| *ACX60_10775* | | *A1S_1475* | *purT* | -2.640385662 | -2.3079 | 0.874075489 | 0.000009 |
| *ACX60_17695* | | *A1S_0076* | *acnD* | -2.920086314 | -2.30352 | 0.788855023 | 0.000957 |
| *ACX60_06990* | | *A1S_2114* | *mutL* | -2.932208549 | -2.3022 | 0.785140985 | 0.000003 |
| *ACX60_16235* | | *A1S_0375* | *corC* | -2.225529464 | -2.24569 | 1.009058951 | 0.171385 |
| *ACX60_10490* | | *A1S_1523* | *-* | -1.648007734 | -2.21597 | 1.344634184 | 0.918702 |
| *ACX60_00705* | | *A1S_3348* | *-* | 0.534220019 | -2.18804 | -4.095757077 | 0.677155 |
| *ACX60_17360* | | *A1S_0144* | *znuC* | -0.402002534 | -2.18782 | 5.442312561 | 0.000008 |
| *ACX60_04845* | | *A1S_2545* | *BCP, PRXQ, DOT5* | -2.163094328 | -2.18631 | 1.010731763 | 0.09045 |
| *ACX60_05320* | | *A1S_2451* | *-* | -1.825325195 | -2.17324 | 1.190602921 | 0.519338 |
| *ACX60_04865* | | *A1S_2540* | *queE* | -2.511166724 | -2.16894 | 0.86371709 | 0.010033 |
| *ACX60_04860* | | *A1S_2542* | *queC* | -2.664683087 | -2.14385 | 0.804541078 | 0.000179 |
| *ACX60_12200* | | *A1S_1205* | *PRDX2_4, ahpC* | -2.103267319 | -2.13856 | 1.016782097 | 0.328522 |
| *ACX60_15830* | | *A1S_0455* | *msrA* | -1.836721983 | -2.13161 | 1.160552097 | 0.08323 |
| *ACX60_09815* | | *A1S_1625* | *E4.6.1.1* | -1.826644816 | -2.11571 | 1.158249769 | 0.039167 |
| *ACX60_15010* | | *A1S_0622* | *mlaA, vacJ* | -0.537078652 | -2.10407 | 3.917617382 | <0.000001 |
| *ACX60_04605* | | *A1S_2591* | *tolQ* | -0.364073301 | -2.10392 | 5.778828567 | 0.000146 |
| *ACX60_01010* | | *A1S_3288* | *-* | -1.445317067 | -2.10267 | 1.454815972 | 0.267849 |
| *ACX60_00395* | | *A1S_3403* | *hutI, AMDHD1* | -2.46391172 | -2.0903 | 0.848364977 | 0.32645 |
| *ACX60_06210* | | *A1S_2268* | *K07018* | -2.126234276 | -2.0847 | 0.980463884 | 0.482802 |
| *ACX60_11795* | | *A1S_1270* | *bccA* | -1.850744176 | -2.0787 | 1.123167666 | 0.012356 |
| *ACX60_05405* | | *A1S_3808* | *-* | -2.274113942 | -2.07862 | 0.91403487 | 0.113295 |
| *ACX60_08615* | | *A1S_1840* | *-* | -1.382210526 | -2.07495 | 1.501184095 | 0.000064 |
| *ACX60_11195* | | *-* | *-* | -2.255831844 | -2.0716 | 0.918329247 | <0.000001 |
| *ACX60_04980* | | *A1S_2517* | *recO* | -1.974569302 | -2.05565 | 1.04106232 | 0.005795 |
| *ACX60_09455* | | *A1S_1690* | *-* | -1.965119091 | -2.04946 | 1.04291911 | 0.38765 |
| *ACX60_17790* | | *A1S_0058* | *gtr6* | -2.313321201 | -2.04478 | 0.883914712 | 0.000689 |
| *ACX60_12035* | | *-* | *-* | -1.966339384 | -2.02766 | 1.031185337 | 0.6735 |
| *ACX60_13810* | | *A1S_3576* | *-* | -2.2972077 | -2.02232 | 0.880337387 | 0.912078 |
| *ACX60_07450* | | *A1S_3772* | *-* | -1.432956287 | -1.98506 | 1.385289114 | 0.127793 |
| *ACX60_01835* | | *A1S_3129* | *astB* | -1.815941045 | -1.97943 | 1.090031517 | 0.384453 |
| *ACX60_05345* | | *A1S_2446* | *pstA* | -2.011887279 | -1.96044 | 0.974427167 | 0.793835 |
| *ACX60_11865* | | *A1S_1258* | *-* | -1.925544261 | -1.95715 | 1.01641294 | 0.275172 |
| *ACX60_13385* | | *A1S_0999* | *wzi* | -0.78292617 | -1.94026 | 2.478217907 | 0.000092 |
| *ACX60_13700* | | *A1S_0936* | *-* | -1.669770044 | -1.9335 | 1.15794383 | 0.006498 |
| *ACX60_08415* | | *A1S_1883* | *aroD* | -2.016614403 | -1.92091 | 0.952542857 | 0.670619 |
| *ACX60_10655* | | *A1S_1496* | *-* | -2.497139968 | -1.9191 | 0.768520844 | 0.214488 |
| *ACX60_04535* | | *A1S_2604* | *-* | -1.852730833 | -1.91885 | 1.035687859 | <0.000001 |
| *ACX60_05340* | | *A1S_2447* | *pstC* | -1.981681847 | -1.91658 | 0.967149345 | 0.000186 |
| *ACX60_16890* | | *A1S_0239* | *E1.1.1.3* | -2.268197392 | -1.90988 | 0.842024592 | 0.000035 |
| *ACX60_13320* | | *A1S_1013* | *ureB* | -1.92737286 | -1.89948 | 0.985529877 | 0.252129 |
| *ACX60_05780* | | *A1S_2350* | *ubiC* | -2.468294356 | -1.89931 | 0.769481335 | 0.003771 |
| *ACX60_01425* | | *A1S_3213* | *-* | -1.987101272 | -1.8905 | 0.951385275 | 0.249145 |
| *ACX60_05010* | | *A1S_2511* | *-* | -1.56083642 | -1.88848 | 1.209914595 | 0.059149 |
| *ACX60_09275* | | *A1S_1726* | *aspA* | -4.333167924 | -1.88628 | 0.435313011 | 0.000001 |
| *ACX60_02245* | | *A1S_3071* | *RP-L14, MRPL14, rplN* | -2.303005484 | -1.88255 | 0.817430731 | 0.865909 |
| *ACX60_03475* | | *A1S_2806* | *elsL* | -1.596103584 | -1.88097 | 1.178475315 | 0.000072 |
| *ACX60_06415* | | *A1S_2227* | *-* | -1.816647235 | -1.88058 | 1.035191241 | 0.005608 |
| *ACX60_15190* | | *A1S_0588* | *rapZ* | -1.772243446 | -1.87086 | 1.055645448 | 0.000005 |
| *ACX60_09335* | | *A1S_1715* | *acpD, azoR* | -1.63243461 | -1.87044 | 1.145796851 | 0.600409 |
| *ACX60_13875* | | *A1S_0902* | *K06996* | -1.560222325 | -1.8699 | 1.198482404 | 0.634043 |
| *ACX60_13285* | | *A1S_1019* | *rsmD* | -1.631867048 | -1.84908 | 1.133106113 | 0.000049 |
| *ACX60_08535* | | *A1S_1857* | *-* | -1.459716393 | -1.84906 | 1.266724194 | 0.887412 |
| *ACX60_17380* | | *A1S_0140* | *ME2, sfcA, maeA* | -2.688917739 | -1.8434 | 0.685555824 | <0.000001 |
| *ACX60_01050* | | *A1S_3280* | *gabD* | -2.679034558 | -1.82809 | 0.682369757 | 0.000061 |
| *ACX60_10960* | | *A1S_1434* | *cydA* | -0.573052997 | -1.82074 | 3.177256578 | 0.000019 |
| *ACX60_10405* | | *A1S_1539* | *-* | -1.803026027 | -1.81563 | 1.006992692 | 0.165052 |
| *ACX60_00480* | | *A1S_3386* | *serB, PSPH* | -2.196314923 | -1.79962 | 0.819381101 | 0.102444 |
| *ACX60_11135* | | *A1S_1398* | *ABC.PA.A* | -1.937793149 | -1.79487 | 0.926241852 | 0.013688 |
| *ACX60_05300* | | *A1S_2455* | *-* | -0.971445435 | -1.78241 | 1.83480306 | 0.384687 |
| *ACX60_11820* | | *A1S_1266* | *-* | -2.031867611 | -1.77788 | 0.874998481 | 0.084892 |
| *ACX60_16350* | | *A1S_0349* | *ubiJ* | -2.072829293 | -1.77655 | 0.857064302 | 0.000031 |
| *ACX60_08305* | | *A1S_1906* | *-* | -2.031547563 | -1.77383 | 0.873142923 | 0.282069 |
| *ACX60_08005* | | *A1S_3747* | *-* | -1.642183905 | -1.76294 | 1.073531529 | 0.063865 |
| *ACX60_15610* | | *A1S_0500* | *pilF* | -2.780094993 | -1.74781 | 0.628688147 | 0.000025 |
| *ACX60_09055* | | *A1S_3726* | *-* | -1.995208491 | -1.7436 | 0.873895837 | 0.315117 |
| *ACX60_11740* | | *-* | *-* | -1.820425995 | -1.72953 | 0.950068632 | 0.000016 |
| *ACX60_11295* | | *A1S_1373* | *E6.4.1.4A* | -2.443207494 | -1.7193 | 0.703707185 | 0.005351 |
| *ACX60_04310* | | *A1S_3824* | *-* | -1.504990583 | -1.71371 | 1.138687275 | 0.004702 |
| *ACX60_02570* | | *A1S_3874* | *clsC* | -0.447618676 | -1.68455 | 3.763354169 | <0.000001 |
| *ACX60_02960* | | *A1S_2907* | *-* | -1.924563395 | -1.68083 | 0.873354747 | 0.030447 |
| *ACX60_11570* | | *A1S_1318* | *-* | -1.437910047 | -1.67631 | 1.165797828 | 0.471012 |
| *ACX60_09460* | | *A1S_1689* | *-* | -1.501087548 | -1.67473 | 1.115677324 | 0.002006 |
| *ACX60_01925* | | *A1S_3113* | *-* | -1.947144586 | -1.67426 | 0.859851633 | 0.427398 |
| *ACX60_01315* | | *A1S_3233* | *-* | -1.913804521 | -1.67421 | 0.874807192 | 0.494722 |
| *ACX60_01830* | | *A1S_3130* | *astD* | -1.714173786 | -1.67397 | 0.976547779 | 0.903811 |
| *ACX60_06250* | | *A1S_2261* | *cspA* | -1.412839021 | -1.67343 | 1.184442341 | 0.00001 |
| *ACX60_13280* | | *A1S_1020* | *mrdA* | -2.028454611 | -1.67013 | 0.82335324 | 0.000005 |
| *ACX60_06960* | | *A1S_3786* | *-* | -1.818248895 | -1.6693 | 0.918081528 | 0.279302 |
| *ACX60_08170* | | *A1S_1929* | *K07078* | -1.562777581 | -1.66612 | 1.066129714 | 0.159058 |
| *ACX60_05970* | | *A1S_2313* | *queF* | -3.437158559 | -1.6619 | 0.483510636 | 0.000099 |
| *ACX60_13215* | | *A1S_1034* | *MTHFS* | -1.410505236 | -1.66052 | 1.177248491 | 0.000491 |
| *ACX60_08820* | | *A1S_1807* | *K03710* | -1.229766472 | -1.65077 | 1.342348245 | 0.000116 |
| *ACX60_04540* | | *A1S_2603* | *hda* | -1.139557358 | -1.64936 | 1.447365791 | 0.002625 |
| *ACX60_07220* | | *A1S_2072* | *-* | -1.396518861 | -1.64366 | 1.176972484 | 0.001326 |
| *ACX60_07865* | | *A1S_1985* | *-* | -2.277079549 | -1.63289 | 0.717099141 | 0.012375 |
| *ACX60_03470* | | *A1S_2807* | *K09790* | -1.437502142 | -1.63209 | 1.135366598 | 0.052624 |
| *ACX60_03425* | | *A1S_2815* | *pilG* | -1.965999432 | -1.61749 | 0.822729272 | 0.809247 |
| *ACX60_07530* | | *A1S_2031* | *-* | -0.873786222 | -1.59996 | 1.831066655 | 0.12865 |
| *ACX60_02390* | | *A1S_3026* | *E3.1.27.1* | -1.285490533 | -1.59364 | 1.239712326 | 0.129527 |
| *ACX60_14470* | | *A1S_0779* | *-* | -1.920998722 | -1.58758 | 0.826435055 | 0.772943 |
| *ACX60_02540* | | *A1S_2997* | *apaH* | -1.296496257 | -1.58321 | 1.221144427 | 0.000575 |
| *ACX60_06455* | | *A1S_2221* | *gltS* | -1.56258166 | -1.56253 | 0.999966066 | 0.000007 |
| *ACX60_16755* | | *A1S_0268* | *hns* | -1.069001015 | -1.56064 | 1.459902062 | 0.010053 |
| *ACX60_08355* | | *A1S_1897* | *-* | -1.196856837 | -1.55849 | 1.302151708 | 0.000163 |
| *ACX60_04875* | | *A1S_2538* | *carO?* | -0.427387829 | -1.5523 | 3.632075671 | 0.203711 |
| *ACX60_12020* | | *A1S_1232* | *-* | -1.395672872 | -1.54459 | 1.106699696 | 0.335312 |
| *ACX60_14880* | | *A1S_0703* | *frmB, ESD, fghA* | -1.915998571 | -1.54225 | 0.804931752 | 0.00698 |
| *ACX60_10280* | | *A1S_1563* | *gspI* | -1.438081467 | -1.52725 | 1.062001815 | 0.000415 |
| *ACX60_10450* | | *A1S_1530* | *putP* | -1.605007239 | -1.52094 | 0.947621535 | 0.079107 |
| *ACX60_07995* | | *-* | *-* | -2.507911783 | -1.51618 | 0.604556831 | 0.150266 |
| *ACX60_10175* | | *A1S_3685* | *-* | -1.232742869 | -1.51549 | 1.229368224 | 0.143383 |
| *ACX60_11275* | | *A1S_1377* | *-* | -1.687626243 | -1.51154 | 0.895661056 | 0.019253 |
| *ACX60_05240* | | *A1S_2466* | *-* | -1.366551375 | -1.48139 | 1.084032897 | 0.023737 |
| *ACX60_07075* | | *A1S_2097* | *-* | -1.360808207 | -1.47947 | 1.087198145 | 0.68343 |
| *ACX60_11285* | | *A1S_1375* | *E6.4.1.4B* | -2.183440171 | -1.47799 | 0.676907016 | 0.00001 |
| *ACX60_09990* | | *-* | *-* | -1.086870157 | -1.47722 | 1.35915297 | 0.766475 |
| *ACX60_10520* | | *A1S_1518* | *fxsA* | -0.907196363 | -1.4768 | 1.627874446 | 0.525152 |
| *ACX60_01125* | | *A1S_3265* | *-* | -1.802256895 | -1.47461 | 0.818203622 | 0.392709 |
| *ACX60_09610* | | *A1S_1666* | *-* | 0.954665885 | -1.46904 | -1.538797482 | 0.602227 |
| *ACX60_13155* | | *A1S_1046* | *lysE, argO* | -1.239618997 | -1.46537 | 1.1821135 | 0.045132 |
| *ACX60_10645* | | *A1S_1498* | *-* | -1.533018545 | -1.46158 | 0.953400518 | 0.305799 |
| *ACX60_07300* | | *A1S_2058* | *-* | -0.680538873 | -1.45943 | 2.14452193 | 0.000013 |
| *ACX60_10525* | | *A1S_1517* | *-* | -1.797747841 | -1.45593 | 0.809864528 | 0.90884 |
| *ACX60_05645* | | *A1S_2380* | *entB, dhbB, vibB, mxcF* | -0.820465773 | -1.4453 | 1.761561623 | 0.337512 |
| *ACX60_03610* | | *A1S_2780* | *mreC* | -1.786284422 | -1.43595 | 0.803874256 | 0.004704 |
| *ACX60_17960* | | *A1S_0025* | *rutR* | -1.077780445 | -1.42381 | 1.321059549 | 0.000061 |
| *ACX60_15360* | | *A1S_0552* | *-* | -1.021262 | -1.42232 | 1.392711387 | 0.000485 |
| *ACX60_07615* | | *A1S_3761* | *-* | -1.366788784 | -1.41984 | 1.038813599 | 0.020211 |
| *ACX60_01275* | | *A1S_3241* | *-* | -1.515054669 | -1.4165 | 0.934949344 | 0.879071 |
| *ACX60_01520* | | *A1S_3196* | *mrcA, pbp1a* | -1.133325802 | -1.41337 | 1.247098723 | 0.940802 |
| *ACX60_13015* | | *A1S_1073* | *prtC* | -1.491802193 | -1.39943 | 0.938080841 | 0.128197 |
| *ACX60_05590* | | *A1S_2392* | *-* | -1.468937255 | -1.38989 | 0.946187976 | 0.000014 |
| *ACX60_16475* | | *A1S_0324* | *rlmB* | -1.504532836 | -1.3862 | 0.921348985 | 0.004749 |
| *ACX60_13920* | | *A1S_0893* | *K09801* | -1.544911052 | -1.38348 | 0.895507339 | 0.002159 |
| *ACX60_02350* | | *A1S_3035* | *xpt* | -0.659408862 | -1.37946 | 2.091966032 | 0.067198 |
| *ACX60_07130* | | *A1S_2086* | *-* | -0.754813857 | -1.37176 | 1.81735532 | 0.00021 |
| *ACX60_01610* | | *-* | *bfd* | -1.154229117 | -1.36618 | 1.183627585 | 0.141151 |
| *ACX60_06740* | | *A1S_2165* | *-* | -1.476753149 | -1.36044 | 0.921236416 | 0.000402 |
| *ACX60_13710* | | *A1S_0934* | *cog0523* | 0.60638037 | -1.35772 | -2.239063522 | 0.025768 |
| *ACX60_11270* | | *A1S_1378* | *K00666* | -1.18647516 | -1.35727 | 1.143950037 | 0.265514 |
| *ACX60_13615* | | *A1S_0954* |  | -1.398450479 | -1.35612 | 0.969730045 | 0.057672 |
| *ACX60_11675* | | *A1S_1295* | *impC* | -1.020402802 | -1.35016 | 1.323161372 | 0.896882 |
| *ACX60_14950* | | *A1S_0688* | *hisC* | -1.479504699 | -1.34835 | 0.911354484 | 0.111729 |
| *ACX60_05665* | | *A1S_2375* | *-* | -0.252373559 | -1.3416 | 5.315935852 | 0.387536 |
| *ACX60_09060* | | *A1S_1761* | *-* | -0.985842101 | -1.33754 | 1.356747784 | 0.387193 |
| *ACX60_09415* | | *A1S_1698* | *lipA* | -1.202535779 | -1.33531 | 1.110412686 | 0.285649 |
| *ACX60_05265* | | *A1S_2462* | *hepA* | -1.599035727 | -1.33096 | 0.83235015 | 0.043774 |
| *ACX60_02645* | | *A1S_2947* | *trxC* | -1.415364704 | -1.32093 | 0.933280382 | 0.000028 |
| *ACX60_12310* | | *A1S_1182* | *crp* | -0.844380487 | -1.32046 | 1.563817807 | 0.000015 |
| *ACX60_11170* | | *A1S_1391* | *-* | -1.373779963 | -1.31883 | 0.959999586 | 0.539524 |
| *ACX60_08885* | | *A1S_1792* | *denD* | -0.997793314 | -1.31873 | 1.321649812 | 0.000328 |
| *ACX60_06965* | | *A1S_2120* | *rsuA* | -1.36325659 | -1.31481 | 0.964461799 | 0.15315 |
| *ACX60_08780* | | *A1S_3733* | *-* | -1.084039376 | -1.31414 | 1.212260508 | 0.038178 |
| *ACX60_15730* | | *A1S_0474* | *fiu* | -2.169707491 | -1.30535 | 0.60162409 | <0.000001 |
| *ACX60_15310* | | *A1S_0562* | *dusA* | -1.327438599 | -1.3028 | 0.981439863 | 0.914697 |
| *ACX60_09600* | | *A1S_1667* | *TC.FEV.OM1, fhuE, fpvA, fptA* | 0.92021597 | -1.30024 | -1.412968056 | <0.000001 |
| *ACX60_00470* | | *A1S_3388* | *ribBA* | -2.527078064 | -1.29832 | 0.513764218 | 0.56805 |
| *ACX60_05145* | | *A1S_2483* | *-* | -0.965793672 | -1.2898 | 1.335484146 | <0.000001 |
| *ACX60_06620* | | *A1S_2192* | *metN* | -1.464032844 | -1.28776 | 0.879600046 | 0.145142 |
| *ACX60_10925* | | *A1S_1443* | *tauB* | -1.103797374 | -1.28495 | 1.164120731 | 0.000378 |
| *ACX60_07990* | | *A1S_1960* | *yrfG* | -1.1261718 | -1.28236 | 1.138688843 | 0.91962 |
| *ACX60_15645* | | *A1S_0492* | *nagZ* | -1.229718551 | -1.28159 | 1.042181721 | <0.000001 |
| *ACX60_05625* | | *A1S_2385* | *TC.FEV.OM* | 0.56875293 | -1.28155 | -2.253257176 | <0.000001 |

**AVG LB** refers to the average log2 fitness of WT in LB. The average includes all 6 biological replicates.

**AVG TPEN** refers to the average log2 fitness of WT in TPEN. The average includes all 6 biological replicates.

**AVG TPEN/LB** creates a comparison between LB and TPEN by dividing **AVG TPEN / AVG LB**.

**Table S4:** Genes with altered fitness in the Δ*zigA* library compared to WT during zinc depletion.

| **Locus Tag**  **(*ACX60_*)** | **Locus Tag**  **(*A1S_*)** | **Gene** | **Avg. Log_2_ Fitness *zigA* (TPEN/LB)** | **Avg. Log_2_ Fitness WT (TPEN/LB)** | **P-value** |
| --- | --- | --- | --- | --- | --- |
| *ACX60_14465* | *A1S_0780* | *mrp, NUBPL* | -0.884640997 | -4.864387228 | 0.000081 |
| *ACX60_09130* | *A1S_1750* | *adeB* | -3.969437182 | -4.806875389 | 0.044827 |
| *ACX60_05430* | *A1S_2427* | *ampG* | -1.884491295 | -4.107067631 | 0.034145 |
| *ACX60_01990* | *A1S_3101* | *mlaD, linM* | -1.776897158 | -3.061603193 | 0.019293 |
| *ACX60_15955* | *A1S_0430* | *lpsB* | -1.658291299 | -3.026289843 | 0.070991 |
| *ACX60_05950* | *A1S_2317* | *rlpA* | -1.32558322 | -2.936775711 | 0.316799 |
| *ACX60_01995* | *A1S_3100* | *mlaC* | -1.257962853 | -2.835531185 | 0.243474 |
| *ACX60_09605* | *-* | *-* | -1.938886918 | -2.724739172 | 0.492413 |
| *ACX60_15920* | *A1S_0438* | *-* | 0.071694494 | -2.504382145 | 0.19088 |
| *ACX60_17365* | *A1S_0143* | *znuB* | -1.310008796 | -2.387859574 | 0.366682 |
| *ACX60_16050* | *A1S_0414* | *nudH* | -0.461364302 | -2.352310075 | 0.188512 |
| *ACX60_09615* | *A1S_3712* | *-* | -1.328716496 | -2.116640014 | 0.283002 |
| *ACX60_15640* | *A1S_0493* | *prc* | -0.991231938 | -2.111005931 | 0.020525 |
| *ACX60_05605* | *A1S_2389* | *ABC.FEV.P* | -1.272929025 | -2.090869281 | 0.010727 |
| *ACX60_04600* | *A1S_2592* | *tolR* | -1.059862455 | -2.063548239 | 0.049828 |
| *ACX60_05620* | *A1S_2386* | *ABC.FEV.S* | -1.301131516 | -2.028896315 | 0.022278 |
| *ACX60_11235* | *A1S_3659* | *-* | -0.243117055 | -1.913318122 | 0.379617 |
| *ACX60_05610* | *A1S_2388* | *ABC.FEV.P* | -0.789738511 | -1.879308141 | 0.019249 |
| *ACX60_05625* | *A1S_2385* | *TC.FEV.OM* | -1.217251497 | -1.8517081 | 0.008502 |
| *ACX60_09670* | *-* | *-* | 0.242284026 | -1.843821379 | 0.102008 |
| *ACX60_15950* | *A1S_0431* | *lpxL, htrB* | -0.822347679 | -1.808499614 | 0.039391 |
| *ACX60_17360* | *A1S_0144* | *znuC* | -1.053537233 | -1.794542181 | 0.053542 |
| *ACX60_17775* | *A1S_0059* | *gtr8* | 0.343840814 | -1.452722988 | 0.283844 |
| *ACX60_03005* | *A1S_2900* | *-* | -0.438188785 | -1.429183801 | 0.002561 |
| *ACX60_03340* | *A1S_2832* | *ppiC* | -0.491518588 | -1.356086644 | 0.681161 |
| *ACX60_17990* | *A1S_0018* | *slpA* | 0.422259007 | -1.281869245 | 0.054476 |
| *ACX60_15075* | *A1S_0610* | *proC* | -0.084792392 | -1.257111842 | 0.305564 |
| *ACX60_09465* | *A1S_1688* | *K09902* | 0.278205689 | -1.241351957 | 0.03933 |
| *ACX60_03825* | *A1S_2737* | *adeK* | 0.470281601 | -1.217499951 | 0.001428 |
| *ACX60_13385* | *A1S_0999* | *wzi* | -0.402538446 | -1.173846569 | 0.006992 |
| *ACX60_04875* | *A1S_2538* | *carO* | -0.236978678 | -1.155484489 | 0.012858 |
| *ACX60_11555* | *A1S_1320* | *soxR* | 0.398772277 | -1.154817295 | 0.199004 |
| *ACX60_13550* | *A1S_3583* | *-* | 0.791300738 | -1.150695109 | 0.050763 |
| *ACX60_15625* | *A1S_0497* | *-* | 0.834759292 | -1.007472945 | 0.013768 |
| *ACX60_00290* | *A1S_3425* | *purC* | 0.986409389 | -0.882042559 | 0.00225 |
| *ACX60_15660* | *A1S_0489* | *proA* | 0.284655387 | -0.86030452 | 0.000683 |
| *ACX60_05300* | *A1S_2455* | *-* | 0.505277076 | -0.823483676 | 0.102315 |
| *ACX60_00700* | *A1S_3349* | *-* | 0.281512644 | -0.74700736 | 0.063585 |
| *ACX60_02350* | *A1S_3035* | *xpt* | 0.374240067 | -0.730279184 | 0.067197 |
| *ACX60_16695* | *-* |  | 0.792037248 | -0.728873608 | 0.001045 |
| *ACX60_08615* | *A1S_1840* | *-* | 0.021689028 | -0.697802988 | 0.027932 |
| *ACX60_10520* | *A1S_1518* | *fxsA* | 0.494414369 | -0.689748129 | 0.092051 |
| *ACX60_08710* | *A1S_1820* | *-* | 0.305507271 | -0.658930937 | 0.06228 |
| *ACX60_06235* | *A1S_2263* | *-* | 0.005963401 | -0.651757361 | 0.077495 |
| *ACX60_03040* | *A1S_2894* | *aspS* | 0.258648548 | -0.637629164 | 0.240659 |
| *ACX60_07130* | *A1S_2086* | *-* | 0.553809626 | -0.629074493 | 0.000034 |
| *ACX60_03015* | *A1S_2898* | *-* | 0.240890992 | -0.625446817 | 0.016684 |
| *ACX60_01930* | *A1S_3112* | *-* | 0.023567978 | -0.610038969 | 0.011293 |
| *ACX60_03105* | *A1S_2881* | *SCD, desC* | 0.249044803 | -0.598667692 | 0.015446 |
| *ACX60_07450* | *A1S_3772* | *-* | 0.136525332 | -0.592497313 | 0.25333 |
| *ACX60_17770* | *A1S_0060* | *gtr9* | 0.32314014 | -0.577537156 | 0.000496 |
| *ACX60_08215* | *-* | *-* | 0.127300852 | -0.561000895 | 0.043981 |
| *ACX60_08705* | *A1S_1821* | *-* | 0.137512994 | -0.533168127 | 0.002432 |
| *ACX60_11430* | *A1S_1347* | *paaX* | 0.292926951 | -0.51924652 | 0.066135 |
| *ACX60_02385* | *A1S_3027* | *slt* | 0.143161134 | -0.518199661 | 0.000222 |
| *ACX60_11390* | *A1S_1351* | *katE, CAT, catB, srpA* | 0.09897214 | -0.489257988 | 0.178614 |
| *ACX60_12795* | *A1S_1111* | *K18383* | 0.322059622 | -0.488352654 | 0.006406 |
| *ACX60_10990* | *A1S_1428* | *-* | 0.254015862 | -0.488060289 | 0.022462 |
| *ACX60_08820* | *A1S_1807* | *K03710* | 0.161812514 | -0.479753041 | 0.126075 |
| *ACX60_15360* | *A1S_0552* | *-* | 0.414498595 | -0.46837121 | 0.016878 |
| *ACX60_15270* | *A1S_0570* | *ybeB* | 0.684233911 | -0.434640565 | 0.021513 |
| *ACX60_09060* | *A1S_1761* | *-* | 0.333915333 | -0.428025953 | 0.033736 |
| *ACX60_06835* | *A1S_2147* | *-* | 0.313592904 | -0.415285039 | 0.071413 |
| *ACX60_02800* | *A1S_2931* | *-* | 0.307121441 | -0.40615024 | 0.063817 |
| *ACX60_08535* | *A1S_1857* | *-* | 0.38994449 | -0.396116985 | 0.016301 |
| *ACX60_04055* | *A1S_2692* | *-* | 0.537677014 | -0.394650584 | 0.06868 |
| *ACX60_09405* | *A1S_1700* | *acoB* | 0.310794687 | -0.385396269 | 0.041882 |
| *ACX60_17960* | *A1S_0025* | *rutR* | 0.538250456 | -0.383553539 | 0.005748 |
| *ACX60_08465* | *A1S_1873* | *-* | 0.292674439 | -0.378817194 | 0.014158 |
| *ACX60_11335* | *A1S_1363* | *-* | 0.233853863 | -0.365652613 | 0.034013 |
| *ACX60_08845* | *A1S_1802* | *TC.BAT2* | 0.274853505 | -0.363127283 | 0.044371 |
| *ACX60_17750* | *A1S_0064* | *gpi* | 0.479203227 | -0.360730068 | 0.000017 |
| *ACX60_06035* | *A1S_2301* | *hisQ* | 0.727125476 | -0.350570714 | 0.04764 |
| *ACX60_10410* | *A1S_1538* | *sstT* | 0.269321676 | -0.345768174 | 0.397113 |
| *ACX60_11675* | *A1S_1295* | *impC* | 0.265671067 | -0.344618352 | 0.039879 |
| *ACX60_09565* | *A1S_1674* | *-* | 0.876820616 | -0.341206997 | 0.070341 |
| *ACX60_12845* | *A1S_1101* | *kdgD* | 0.554126873 | -0.336344492 | 0.00009 |
| *ACX60_12630* | *-* | *-* | 0.315256378 | -0.33195229 | 0.005809 |
| *ACX60_12580* | *A1S_1146* | *DNMT1, dcm* | 0.368320665 | -0.327095195 | 0.012538 |
| *ACX60_17740* | *A1S_0066* | *pgm* | 0.393724713 | -0.325293498 | 0.000987 |
| *ACX60_05260* | *A1S_2463* | *rluA* | 0.583851541 | -0.319959517 | 0.006469 |
| *ACX60_12390* | *A1S_3621* | *-* | 0.390110428 | -0.319313483 | 0.068457 |
| *ACX60_18150* | *A1S_2974* | *hprT, hpt, HPRT1* | 0.366386662 | -0.317572164 | 0.062669 |
| *ACX60_13640* | *A1S_0948* | | 0.436561938 | -0.317416476 | 0.000205 |
| *ACX60_00495* | *A1S_3383* | *dtd, DTD1* | 0.345526243 | -0.315019523 | 0.125587 |
| *ACX60_03470* | *A1S_2807* | *K09790* | 0.374523548 | -0.313147106 | 0.115605 |
| *ACX60_02540* | *A1S_2997* | *apaH* | 0.347463103 | -0.312485506 | 0.002409 |
| *ACX60_15830* | *A1S_0455* | *msrA* | 0.604159283 | -0.30993632 | 0.05581 |
| *ACX60_10175* | *A1S_3685* | *-* | 1.003512105 | -0.295144192 | 0.003604 |
| *ACX60_08295* | *A1S_1908* | *E2.5.1.54, aroF, aroG, aroH* | 0.328180761 | -0.285994787 | 0.054693 |
| *ACX60_02505* | *A1S_3004* | *-* | 0.388643406 | -0.285900436 | 0.003861 |
| *ACX60_07320* | *A1S_2054* | *ybgC* | 0.747336104 | -0.278526231 | 0.002213 |
| *ACX60_11155* | *A1S_1394* | *-* | 0.369610374 | -0.274017838 | 0.075456 |
| *ACX60_13840* | *A1S_0909* | *emrB* | 0.359125971 | -0.271657201 | 0.002611 |
| *ACX60_10515* | *-* | *-* | 1.039121523 | -0.266743309 | 0.006326 |
| *ACX60_11885* | *-* | *-* | 0.507867109 | -0.265397367 | 0.032185 |
| *ACX60_07755* |  | *-* | 0.503841239 | -0.262744497 | 0.050951 |
| *ACX60_11500* | *A1S_1332* | *aldH* | 0.346312547 | -0.26182705 | 0.056442 |
| *ACX60_08595* | *A1S_1844* | *catC* | 0.615273092 | -0.26039295 | 0.004545 |
| *ACX60_09110* | *-* | *-* | 0.500017749 | -0.258347971 | 0.017777 |
| *ACX60_13880* | *A1S_0901* | *-* | 0.441280276 | -0.255789128 | 0.024015 |
| *ACX60_00930* | *A1S_3304* | *-* | 0.40880138 | -0.249628319 | 0.013204 |
| *ACX60_01610* | *-* | *bfd* | 0.37169055 | -0.249355657 | 0.14115 |
| *ACX60_12115* | *-* | *-* | 0.378156182 | -0.249167857 | 0.096648 |
| *ACX60_07645* | *-* | *-* | 0.889657585 | -0.244943087 | 0.068755 |
| *ACX60_00870* | *A1S_3316* | *prfB* | 0.42323111 | -0.241892661 | 0.421471 |
| *ACX60_05480* | *A1S_2413* | *-* | 0.423936493 | -0.239989276 | 0.159811 |
| *ACX60_08920* | *A1S_1785* | *ABC.FEV.A* | 0.380068806 | -0.229167273 | 0.185003 |
| *ACX60_05045* | *A1S_2505* | *-* | 0.65817323 | -0.227607349 | 0.023363 |
| *ACX60_01710* | *A1S_3156* | *K07090* | 0.373120759 | -0.220700105 | 0.170633 |
| *ACX60_07290* | *A1S_2060* | *-* | 0.563318441 | -0.214289053 | 0.008009 |
| *ACX60_03360* | *A1S_2828* | *-* | 0.521096278 | -0.209977992 | 0.0222 |
| *ACX60_08645* | *A1S_1833* | *cpo* | 0.42090662 | -0.20734693 | 0.147025 |
| *ACX60_02015* | *-* | *mutT, NUDT15, MTH2* | 0.457057209 | -0.205172085 | 0.008266 |
| *ACX60_10435* | *A1S_1533* | *-* | 0.414820746 | -0.203241074 | 0.217771 |
| *ACX60_15220* | *A1S_0581* | *-* | 0.396864156 | -0.202171446 | 0.053751 |
| *ACX60_02000* | *A1S_3099* | *mlaB* | 0.44489704 | -0.201893777 | 0.02623 |
| *ACX60_17170* | *A1S_0181* | *-* | 1.330205407 | -0.201691458 | 0.00031 |
| *ACX60_12015* | *A1S_3634* | *-* | 0.53498655 | -0.199312366 | 0.069955 |
| *ACX60_03020* | *A1S_3841* | *K07270* | 0.528782393 | -0.195937546 | 0.04022 |
| *ACX60_16170* | *A1S_0389* | *crcB, FEX* | 0.880527242 | -0.191466385 | 0.009217 |
| *ACX60_02380* | *A1S_3028* | *miaB* | 0.812485119 | -0.190279603 | 0.000138 |
| *ACX60_05400* | *A1S_2434* | *-* | 0.527955781 | -0.183867166 | 0.082607 |
| *ACX60_07895* | *A1S_1979* | *adeN* | 0.443623208 | -0.183703647 | 0.004694 |
| *ACX60_00945* | *A1S_3301* | *-* | 0.454645111 | -0.180424454 | 0.029075 |
| *ACX60_13845* | *A1S_0908* | *emrA* | 0.440003497 | -0.169927717 | 0.009321 |
| *ACX60_17050* | *A1S_0207* | *-* | 0.457103284 | -0.169198927 | 0.00492 |
| *ACX60_10685* | *A1S_1490* | *gltI, aatJ* | 0.420047547 | -0.168816354 | 0.01546 |
| *ACX60_01030* | *A1S_3284* | *-* | 0.722512177 | -0.165412322 | 0.014565 |
| *ACX60_05685* | *A1S_2371* | *-* | 0.432093432 | -0.161771517 | 0.001849 |
| *ACX60_08775* | *-* | *-* | 0.523013111 | -0.159858257 | 0.040169 |
| *ACX60_03345* | *A1S_2831* | *mutM, fpg* | 0.505291166 | -0.159797768 | 0.011725 |
| *ACX60_17410* | *A1S_0133* | *-* | 0.544316153 | -0.153472684 | 0.064895 |
| *ACX60_14875* | *A1S_0704* | *K09956* | 0.454115632 | -0.151819171 | 0.067986 |
| *ACX60_16175* | *A1S_0388* | *-* | 0.614059128 | -0.146709014 | 0.062563 |
| *ACX60_17545* | *A1S_0106* | *paaF, echA* | 0.607560126 | -0.143597237 | 0.005752 |
| *ACX60_05240* | *A1S_2466* | *-* | 0.490713776 | -0.142642427 | 0.107891 |
| *ACX60_16190* | *-* | *K09862* | 0.581769237 | -0.142003854 | 0.135194 |
| *ACX60_12565* | *A1S_3611* | *-* | 0.874766424 | -0.14111187 | 0.018758 |
| *ACX60_16070* | *A1S_0410* | *hcaR* | 0.632494205 | -0.139938577 | 0.000303 |
| *ACX60_04245* | *A1S_2655* | *-* | 0.654302766 | -0.139547155 | 0.148357 |
| *ACX60_12615* | *A1S_3606* | *-* | 0.501549722 | -0.139073756 | 0.004785 |
| *ACX60_14255* | *A1S_0823* | *ribB, RIB3* | 0.545767573 | -0.138927271 | 0.000372 |
| *ACX60_11350* | *A1S_1359* | *afuA, fbpA* | 0.620916881 | -0.133862711 | 0.034597 |
| *ACX60_03150* | *A1S_2872* | *K07044* | 0.523418404 | -0.131996119 | 0.006137 |
| *ACX60_10105* | *A1S_3690* | *-* | 0.614582347 | -0.127050241 | 0.008869 |
| *ACX60_16460* | *A1S_0327* | *pilD, pppA* | 0.504466779 | -0.122632543 | 0.00573 |
| *ACX60_14900* | *A1S_0699* | *tsaB* | 0.562555982 | -0.122037972 | 0.018951 |
| *ACX60_11765* | *A1S_1275* | *K09967* | 0.687895626 | -0.119630078 | 0.000583 |
| *ACX60_14175* | *A1S_0840* | *bamD* | 0.665491965 | -0.119329013 | 0.01204 |
| *ACX60_08020* | *A1S_1957* | *KYNU, kynU* | 0.552537882 | -0.118886955 | 0.031654 |
| *ACX60_09170* | *A1S_1743* | *-* | 0.477066231 | -0.115956927 | 0.027322 |
| *ACX60_04535* | *A1S_2604* | *-* | 0.524176981 | -0.114360937 | 0.176981 |
| *ACX60_09560* | *A1S_1675* | *-* | 0.891017665 | -0.112541609 | 0.048939 |
| *ACX60_03390* | *A1S_2822* | *-* | 0.563527591 | -0.110782515 | 0.017905 |
| *ACX60_01130* | *A1S_3893* | *-* | 0.616829024 | -0.104968274 | 0.00002 |
| *ACX60_15090* | *A1S_0607* | *ppx-gppA* | 0.617613168 | -0.096846766 | 0.024587 |
| *ACX60_04060* | *A1S_2691* | *-* | 0.578111174 | -0.096670916 | 0.012962 |
| *ACX60_03310* | *A1S_2838* | *lysP* | 0.583765617 | -0.095595436 | 0.006543 |
| *ACX60_01080* | *A1S_3274* | *-* | 0.635555975 | -0.094457423 | 0.016779 |
| *ACX60_12205* | *A1S_1204* | *-* | 0.576878109 | -0.091912526 | 0.005269 |
| *ACX60_15350* | *A1S_0554* | *prfC* | 0.814208581 | -0.091416876 | 0.000925 |
| *ACX60_12035* | *-* | *-* | 0.528027133 | -0.09089041 | 0.1846 |
| *ACX60_13510* | *A1S_0973* | *dsdA* | 0.529008463 | -0.086877885 | 0.010729 |
| *ACX60_08290* | *A1S_1909* | *TC.SMR3* | 0.565810092 | -0.086212419 | 0.108895 |
| *ACX60_01885* | *-* | *-* | 0.510090614 | -0.085790756 | 0.199986 |
| *ACX60_04335* | *A1S_2644* | *-* | 0.593882723 | -0.080236527 | 0.05217 |
| *ACX60_04090* | *A1S_2685* | *-* | 0.544707941 | -0.080135321 | 0.015642 |
| *ACX60_07315* | *A1S_2055* | *plc* | 0.551575182 | -0.079084551 | 0.017575 |
| *ACX60_10080* | *-* | *-* | 0.89605404 | -0.072319292 | 0.223177 |
| *ACX60_10035* | *-* | *-* | 0.657144247 | -0.053666202 | 0.003404 |
| *ACX60_17370* | *A1S_0142* | *rhtB* | 0.631500069 | -0.049459591 | 0.004277 |
| *ACX60_01285* | *A1S_3239* | *-* | 0.552072841 | -0.048681902 | 0.04514 |
| *ACX60_03915* | *A1S_2703* | *-* | 0.633407996 | -0.045965041 | 0.003182 |
| *ACX60_14545* | *A1S_0767* | *-* | 0.604377919 | -0.042369703 | 0.009956 |
| *ACX60_12695* | *A1S_1132* | *-* | 0.580300591 | -0.039174838 | 0.031691 |
| *ACX60_06940* | *A1S_3787* | *-* | 0.583554758 | -0.033645067 | 0.051352 |
| *ACX60_05030* | *A1S_2508* | *aspB* | 0.720537019 | -0.027757039 | 0.004813 |
| *ACX60_15855* | *A1S_0450* | *-* | 0.853336118 | -0.024618952 | 0.001494 |
| *ACX60_16590* | *A1S_0300* | *-* | 0.707260589 | -0.021688029 | 0.046867 |
| *ACX60_16700* | *A1S_0279* | *tuf, TUFM* | 0.988300558 | -0.021422074 | 0.006523 |
| *ACX60_00925* | *A1S_3305* | *ssuE* | 0.769908379 | -0.02073724 | 0.000225 |
| *ACX60_11800* | *A1S_1269* | *atzF* | 0.672319912 | -0.019739962 | 0.004134 |
| *ACX60_00740* | *A1S_3340* | *pyrE* | 0.648941418 | -0.016995048 | 0.17522 |
| *ACX60_07370* | *A1S_2041* | *-* | 0.636901109 | -0.01535874 | 0.001739 |
| *ACX60_10745* | *A1S_1479* | *-* | 0.60925109 | -0.00412844 | 0.049258 |
| *ACX60_14330* | *A1S_0808* | *bioC* | 1.074581955 | 0.003708257 | 0.007713 |
| *ACX60_17120* | *A1S_0191* | *K09858* | 1.055177189 | 0.016955064 | 0.000005 |
| *ACX60_12835* | *A1S_1103* | *-* | 1.139331014 | 0.040391748 | 0.000002 |
| *ACX60_04865* | *A1S_2540* | *queE* | 1.148005324 | 0.281060422 | 0.043423 |
| *ACX60_15485* | *A1S_0526* | *-* | 1.345487768 | 0.324740915 | 0.065865 |
| *ACX60_02860* | *A1S_3847* | *elsS* | 1.08206524 | 0.33605697 | 0.086846 |
| *ACX60_10340* | *A1S_1551* | *parA, soj* | 1.198920773 | 0.344400049 | 0.051303 |
| *ACX60_10350* | *A1S_1549* | *murU* | 1.360267888 | 0.372184592 | 0.017403 |
| *ACX60_05960* | *A1S_2315* | *rodA, mrdB* | 1.064625976 | 0.428907212 | 0.006629 |
| *ACX60_08195* | *A1S_2313* | *queF* | 1.398056786 | 0.512164634 | 0.000016 |
| *ACX60_15615* | *A1S_0499* | *rlmN* | 1.203363516 | 0.554895937 | 0.008254 |
| *ACX60_04400* | *A1S_2632* | *lysA* | 1.294994196 | 0.584590134 | 0.039335 |
| *ACX60_11285* | *A1S_1375* | *E6.4.1.4B* | 1.445014773 | 0.636606761 | 0.018474 |
| *ACX60_15730* | *A1S_0474* | *fiu* | 1.691804853 | 0.856555867 | 0.000211 |
| *ACX60_00400* | *A1S_3402* | *hutG* | 1.845375542 | 1.021702103 | 0.001233 |
| *ACX60_15880* | *A1S_0445* | *-* | 2.234808667 | 1.258686516 | 0.119885 |

**Avg. Log_2_ Fitness *zigA* (TPEN/LB):** creates a comparison between LB and TPEN by dividing **AVG TPEN / AVG LB** for the Δ*zigA* mutant.

**Avg. Log_2_ Fitness WT (TPEN/LB):** creates a comparison between LB and TPEN by dividing **AVG TPEN / AVG LB** for WT.

**References:**

1. Wijers CDM, Pham L, Menon S, Boyd KL, Noel HR, Skaar EP, Gaddy JA, Palmer LD, Noto MJ. Identification of Two Variants of *Acinetobacter baumannii* Strain ATCC 17978 with Distinct Genotypes and Phenotypes. *Infect Immun*. 2021 Nov 16;89(12):e0045421.
2. Wang N, Ozer EA, Mandel MJ, Hauser AR. Genome-wide identification of *Acinetobacter baumannii* genes necessary for persistence in the lung. *mBio*. 2014 Jun 3;5(3):e01163-14. doi: 10.1128/mBio.01163-14.
3. Hoang TT, Karkhoff-Schweizer RR, Kutchma AJ, Schweizer HP. A broad-host-range Flp-FRT recombination system for site-specific excision of chromosomally-located DNA sequences: application for isolation of unmarked *Pseudomonas aeruginosa* mutants. Gene. 1998 May 28;212(1):77-86.
4. Ménard R, Sansonetti PJ, Parsot C. Nonpolar mutagenesis of the ipa genes defines IpaB, IpaC, and IpaD as effectors of *Shigella flexneri* entry into epithelial cells. *J Bacteriol*. 1993 Sep;175(18):5899-906.
5. Figurski DH, Helinski DR. Replication of an origin-containing derivative of plasmid RK2 dependent on a plasmid function provided in trans. *Proc Natl Acad Sci U S A*. 1979 Apr;76(4):1648-52.
6. Choi KH, Gaynor JB, White KG, Lopez C, Bosio CM, Karkhoff-Schweizer RR, Schweizer HP. A Tn7-based broad-range bacterial cloning and expression system. *Nat Methods.* 2005 Jun;2(6):443-8. doi: 10.1038/nmeth765. PMID: 15908923.
7. Carruthers, M. D., Nicholson, P. A., Tracy, E. N., & Munson, R. S. (2013). Acinetobacter baumannii utilizes a type VI secretion system for bacterial competition. PLoS ONE, 8(3).
